# Do Language Models Know Who Did What to Whom?

**DOI:** 10.1162/OPMI.a.365

**Published:** 2026-07-07

**Authors:** Joseph M. Denning, Xiaohan (Hannah) Guo, Bryor Snefjella, Idan A. Blank

**Affiliations:** Department of Psychology, University of California, Los Angeles, Los Angeles, CA, USA; Department of Psychology, University of Chicago, Chicago, IL, USA

**Keywords:** language models, comprehension, thematic roles, representational similarity

## Abstract

Language models (LMs) are commonly criticized for not “understanding” language. However, many critiques focus on cognitive abilities that, in humans, are distinct from language processing. Here, we instead study a kind of understanding tightly linked to language: inferring “who did what to whom” (thematic roles) in a sentence. Does the central training objective of LMs—word prediction—result in sentence representations that capture thematic roles? In two experiments, we characterized sentence representations in four LMs that have been proposed as models of human language processing. The overall representational similarity of sentence pairs did not reflect whether they had the same agent/patient assignments or opposite agent/patient assignments. Furthermore, we found limited evidence that thematic role information was available in any subspace of hidden activations. However, some attention heads robustly captured thematic roles, independently of syntax. Therefore, LMs can extract thematic roles but this information influences their representations weakly.

## INTRODUCTION

Language Models (LMs) have achieved unprecedented success at natural language processing. Their success demonstrates the power of statistical learning over strings of linguistic forms (Contreras Kallens et al., 2023; Piantadosi, 2023): by merely learning to predict the next word (or a missing word) in a text, LMs develop the ability to produce texts that conform to the syntactic rules of a language (e.g., McCoy et al., 2023). Indeed, the internal representations and next-word predictions of LMs suggest that these systems have acquired many complex grammatical generalizations (for reviews, see Chang & Bergen, 2024; Futrell & Mahowald, 2026; Linzen & Baroni, 2021). As a result, LMs have been proposed as models of human language processing (Blank, 2023) and are being used to predict both behavioral and neuroimaging data (Antonello et al., 2021; Caucheteux & King, 2022; Evanson et al., 2023; Hosseini et al., 2022; Merkx & Frank, 2020; Oh & Schuler, 2022; Schrimpf et al., 2021; Tang et al., 2023; Tuckute et al., 2024; Wilcox et al., 2020). Nonetheless, humans do not use grammar as an end in itself, but rather as an intermediate step in mapping linguistic input forms (e.g., phonological or orthographic information) onto meaning (semantics; Jackendoff & Wittenberg, 2017). Therefore, a key question is whether LMs can extract meaning from their textual input. More specifically: what kinds of meaning can be acquired merely from learning to predict the next (or a missing) word?

The general question of semantics in LMs is hotly debated. Whereas the behavior and internal activity of LMs exhibit some signatures of semantic representations (for a review, see Chang & Bergen, 2024; Pavlick, 2022), these models are often criticized for not truly “understanding” language (e.g., Bender & Koller, 2020). The critiques use various definitions of “understanding”. Some critics claim that LMs do not have “grounded” knowledge that links linguistic meaning to non-linguistic experience (Bender & Koller, 2020; Bisk et al., 2020; Merrill et al., 2021); others claim that LMs lack common sense, i.e., intuitive theories about how the world works (Sinha et al., 2019; Ullman, 2023); yet others claim that LMs do not reason logically (Ettinger, 2020; Wu et al., 2023). All such criticisms conclude that LMs do not understand language like humans do.

However, these critiques are valid only to the extent that they rely on accurate notions of language comprehension in humans. Humans are, of course, able to relate linguistic input to non-linguistic experiences, evaluate such input against their common sense and prior knowledge, and use it for logical inferences. But these capacities are kinds of “thinking”, not language (Mahowald et al., 2024): in the human mind, the cognitive systems that support sensorimotor or affective processes, common sense reasoning, and logical inferences are functionally distinct from the system that analyzes linguistic input (Fedorenko et al., 2024; Fedorenko & Varley, 2016). Whereas linguistic processing is a prerequisite for, e.g., evaluating whether a sentence is consistent with common sense, these two processes are dissociable, and a failure to carry out the latter does not demonstrate a failure to carry out the former (by analogy, if a human fails to paint a copy of a picture, that does not mean they failed to visually perceive that picture). Given that the mind appears to dedicate a system to linguistic processing per se, a fair yet critical bar for LMs to pass is semantic processing that is “language-internal” (e.g., Piantadosi & Hill, 2022, but see Jackendoff, 2012).

In focusing on “language-internal” understanding, we do not suggest that testing LMs on “language-external” thinking and reasoning is in general misguided. Both types of comprehension are important, and a full understanding of LMs would require mapping the entire computational trajectory from forms, through linguistic meaning, and onto complex aspects of thought that these systems may be capable of. But our goal here is to evaluate LMs as models of human language (rather than computational systems in their own right). From this perspective, the distinction between “language-internal” and “language-external” aspects of comprehension is critical, especially given that many aspects of “language-external” thinking are not acquired primarily through language, and might precede language during development. Critiquing LMs for failing to think is different from critiquing them for failing to understanding language “per se”. Therefore, here we test a case of understanding that is “language-internal” or, at least, closely tied to language.

Specifically, we test whether LMs can use the structure of a sentence that describes a simple event to figure out “who did what to whom”, a process called “thematic role assignment” (Dryer, 2002; Fenk-Oczlon & Fenk, 2008; Fillmore, 1968; Greenberg, 1963; Jackendoff, 1972; Kiparsky, 1997; Koplenig et al., 2017; Levin & Hovav, 2005; Levshina, 2020, 2021; Nakamura et al., 2024; Sinnemäki, 2008; for a review, see Rissman & Majid, 2019). We focus on the mapping from grammatical positions to the thematic roles of an action’s “agent” and “patient” (we use “patient” loosely to refer to themes, goals, and recipients, depending on the stimulus, because they share conceptual similarities and cluster under a “proto-patient” role; Dowty, 1991). This mapping is variable across syntactic structures: in an active sentence like “the poet quoted the diplomat”, the grammatical subject (poet) is the agent, and the grammatical object (diplomat) is the patient; however, in a passive sentence, like “the diplomat was quoted by the poet”, the mapping is different: the diplomat is still the patient, but it is not the grammatical subject, whereas the poet–still the agent, as in the previous, active sentence–is now inside the adjunct. As this example demonstrates, two sentences with different grammatical constructions (active vs. passive) can have the same thematic role assignments. Alternatively, two sentences with the same grammatical construction can have opposite thematic role assignments, e.g., “the poet quoted the diplomat” vs. “the diplomat quoted the poet”. Inferring who did what to whom requires combining several types of syntactic information (linear order, construction), at least in the absence of prior semantic information about which noun is a more plausible agent (Caramazza & Zurif, 1976; Mahowald et al., 2023).

Thematic role assignment is often invoked as an important component of language processing in psycholinguistic theories (for a review, see: Rissman & Majid, 2019), unlike many other tasks used to test LMs, such as logical entailment or common-sense reasoning (e.g., Srivastava et al., 2022; Wang et al., 2018). It is central to the syntax-semantics interface (Goldberg, 1995, 2019) and becomes available rapidly to guide incremental comprehension (Altmann, 1999; Gennari & MacDonald, 2008; Lee & Phillips, 2025). However, it appears to be dissociable from syntactic processing as such (Caramazza & Miceli, 1991; Chatterjee et al., 1995; Ziegler & Snedeker, 2018), and thus the evidence for syntactic representations in LMs does not trivially predict that thematic role assignment would be successful.

In the human brain, thematic role assignment engages the Core Language Network (Ivanova et al., 2021), a system selective for high-level language processing (Fedorenko et al., 2011, 2024), including the extraction of meaning from sentences (Fedorenko et al., 2016; Regev et al., 2024). Whereas thematic role assignment also recruits a-modal regions located outside the Core Language Network, this recruitment critically relies on task demands beyond role assignment itself: unlike the Core Language Network, these other regions are overall not sensitive to linguistic meaning (Ivanova et al., 2025; see also Frankland & Greene, 2020; Wang et al., 2016). Thus, understanding “who did what to whom” in a sentence appears to predominately (or, at least, heavily) rely on computations that are linguistic in nature, and thus provides an appropriate test for LMs.

In two experiments, we test whether training LMs on a word-prediction objective results in representations that reflect thematic roles. To this end, we use LMs that are pre-trained on this objective, without any further fine-tuning on other objectives. Five matters about our rationale are worth emphasizing. First, LMs can be directly trained on the specific task of thematic role assignment (also called “semantic role labeling”) via supervised learning, wherein training sentences are paired with correct role labels for different words. However, our question is whether the broader training objective of word prediction suffices for this purpose. Word prediction is the consensus objective for (pre)training LMs and, as mentioned above, such LMs are treated as “general language processors” that are, e.g., compared to human behavior and brain activity. Our study asks whether these models “understand” sentences in the minimal sense of thematic role assignment. If such LMs do not represent thematic roles in a human-like way, then the variance in human data that these LMs can predict likely does not reflect this basic aspect of meaning.

Second, larger and more recent LMs, like ChatGPT (OpenAI, 2022) or GPT5 (Singh et al., 2026), are not only (pre)trained on word prediction but are also fine-tuned using “reinforcement learning from human feedback” (RLHF; Christiano et al., 2017; Ouyang et al., 2022) or similar methods. In RLHF, an LM receives input about human preferences and learns a policy for generating text that is maximally aligned with those preferences. Such “evaluative” input—information about how humans would like the LM to respond to their queries—constitutes non-linguistic information according to our construal of the distinction between language and thought. LMs trained with RLHF are therefore outside the scope of the current work, given that we are asking whether meaning can be learned just from information about the distribution of words and sentence structures.

Third, prior work has demonstrated that the internal representations of LMs contain information about thematic roles (e.g., Proietti et al., 2022, 2024; Tenney, Das, & Pavlick, 2019; Tenney, Xia, et al., 2019). However, the presence of such information is often tested using corpora derived from natural text (e.g., Carreras & Màrquez, 2005; Pradhan et al., 2013), which likely contains few challenging examples: in many sentences, thematic roles might be assigned based on heuristics or shortcuts (Mahowald et al., 2023). Our study instead relies on stimuli that are specifically designed to be less susceptible to such heuristics: both experiments use “reversible” sentences in which both agent and patient are equally likely to produce the action, and Experiment 2 uses a wide range of grammatical constructions. We chose this approach because other linguistic capacities that appear robust when tested on corpora “from the wild” can break down when tested on carefully crafted stimuli, like those used in controlled psycholinguistic experiments conducted with humans (e.g., Chaves & Richter, 2021; Glockner et al., 2018; McCoy et al., 2019; Rosenman et al., 2020; Sinha et al., 2021).

Fourth, testing whether LMs map syntax onto thematic roles assumes that a representation of syntactic structure is available to LMs. However, syntactic processing in LMs still falls short of humans (Aina & Linzen, 2021; Diego-Simón et al., 2025; Huang et al., 2024; Marvin & Linzen, 2018; van Schijndel & Linzen, 2021; Warstadt & Bowman, 2020; Warstadt et al., 2020; but see Hu et al., 2025); and even when LMs do capture the structure of sentences, they might rely on “tricks” that differ from the rich, systematic linguistic principles guiding humans (Chaves & Richter, 2021; McCoy et al., 2019; Sinha et al., 2021). Still, such characterizations of LMs are often based on sentences with quite complex structures. There is wide implicit agreement that LMs do capture the structure of simple sentences like “the poet quoted the diplomat”, which are the stimuli used in Experiment 1. Additionally, by using controlled stimuli where relying on heuristics vs. syntactic information would lead to distinct representations, some experiments have demonstrated that the internal activity patterns of LMs robustly encode syntactic information such as grammatical roles (Papadimitriou et al., 2022).

Fifth, and finally, our approach involves investigating the internal representations of LMs rather than prompting them (e.g., directly asking the models “what is the agent of this sentence?” or “do these sentences have the same thematic roles?” see Ettinger et al., 2016, 2018). We use this probing approach because a prompting task would require that these models not only assign thematic roles, but also that they understand the question being asked of them and understand what words like “agent” or “thematic roles” mean (meta-linguistic abilities). Probing the models directly allows us to get around these task demands, and previous work shows that these distinct approaches can lead to differing results (Hu & Frank, 2024; Hu & Levy, 2023; Hu et al., 2024). Even if an LM can answer an explicit prompt correctly, such accuracy does not demonstrate that the model has a robust and generalizable internal encoding of thematic roles. Instead, the LM might “simulate” understanding via shallow heuristics, memorization of templates, or other distributional patterns in its training corpora. Moreover, prompts measure performance in a specific task context. Studying LM knowledge across sufficiently many contexts and prompt variations is practically challenging and, moreover, performance that is not stable across such variations would be difficult to interpret (are some prompts “bad” or is the LM brittle?). Rather than quantifying performance via LM outputs, internal representations quantify something closer to the underlying “capacity”: abstract latent structure that is apparent even when not explicitly prompted.

**Figure 1.**
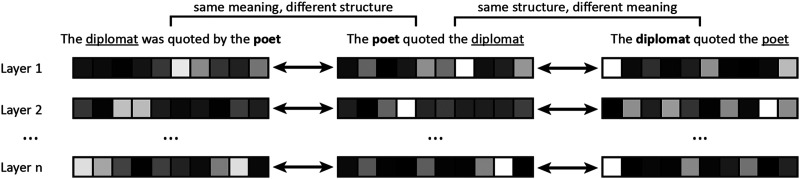
Basic approach of Experiment 1. We feed different sentences (each separately) into a LM and, for each layer, extract the corresponding internal representations, depicted here as squares representing hidden units with different levels of activation (brightness levels). We then compare the representation of sentence pairs that either (i) share thematic role assignments but differ in syntax (left and middle sentences) or (ii) have opposite thematic role assignments but share syntax (middle and right sentences). In each sentence, the agent is bolded and the patient is underlined for illustrative purposes.

**Figure 2.**
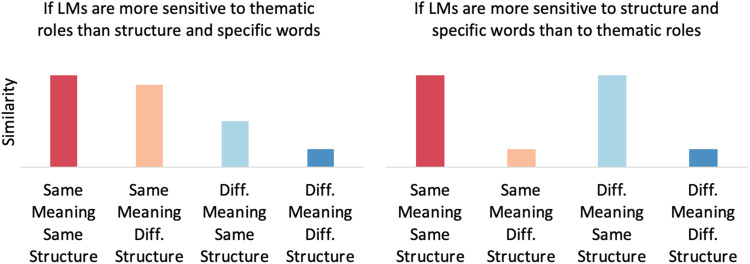
Expected similarity patterns between sentence pairs under two hypotheses: LMs are more sensitive to thematic roles than to structure and specific words (left), or LMs are more sensitive to structure and specific words than to thematic roles (right).

Our approach is therefore appropriate for testing whether LMs implicitly assign roles like “agent” and “patient” to event participants in a sentence. Our main analyses rely on the following paradigm: we feed different sentences to these LMs, extract the resulting sentence representations—in the form of patterns of activity in hidden layers—and quantitatively characterize to what extent they are influenced by thematic role information. Specifically, we generate sentences that either (i) share thematic role assignments but differ in syntax (e.g., “the poet quoted the diplomat” and “the diplomat was quoted by the poet”), or (ii) have opposite thematic role assignments but share syntax (e.g., “the poet quoted the diplomat” and “the diplomat quoted the poet”). We test whether sentence pairs of type (i) are more similar to one another than pairs of type (ii), which would be expected if word prediction suffices for LMs to learn something akin to thematic roles, and if this aspect of meaning plays a larger role than syntax in internal representations (Figures 1, 2). This is a simple analogue to representational similarity analyses in fMRI (Kriegeskorte et al., 2008; it is perhaps most similar to the fMRI method “multi-voxel pattern analysis”, Norman et al., 2006, see other work that uses a similar method: Cong, 2024; Nicolas & Caliskan, 2024).

The test above is stringent: it not only requires that LMs extract thematic role information, but also that this information exerts a stronger influence than syntactic form on the overall organization of representations in a given layer of the LM. Such a test is important insofar as language is a tool for communicating meaning, so any model of human language processing should be expected to give more weight to semantics than to syntax (the latter not being an end in itself). This point can be further clarified with an analogy to static (context-less) word embeddings: such representational spaces implement the “distributional hypothesis” whereby the meaning of a word is influenced by the contexts in which that word occurs (de Saussure, 2011; Firth, 1962; Harris, 1954); therefore, the overall organization of vectors in a static word embedding is generally expected to be governed more strongly by meaning than by aspects of form (e.g., part of speech). Similarly, we may expect that in some layers of a LM, the representational space of sentences would reflect the meaning of those sentences—including thematic role information—more strongly than their form.

In our first experiment, we measure the overall similarity between the LM’s internal representations of sentence pairs (in the hidden units of the "residual stream”). Our stimuli consist of active, transitive sentences (subject + verb + object) and their passive counterparts as in the preceding examples. To foreshadow our results, representations of sentences with opposite thematic roles (but the same active structure) are more similar to one another than representations of sentences with the same thematic roles (but different syntax, i.e., active vs. passive). Because this is a stringent test, we treat the result as preliminary and, in Experiment 2, follow up with more sensitive tests for thematic role information: in addition to testing for overall representational similarity, we ask whether the high-dimensional representational space of the residual stream contains any lower-dimensional subspace that reflects thematic roles, and we also examine representations generated by attention heads. Moreover, we use a wider variety of syntactic structures to test whether the representation of thematic roles is robust.

## EXPERIMENT 1

### Methods

#### Stimuli.

Stimuli were based on a fMRI experiment by Fedorenko et al. (2020) and generated in two steps: First, we created 94 “base” active, transitive sentences describing a two participant event, such as “the lawyer saved the author”. Then, we edited each base sentence to create four versions that changed its thematic role assignments (and hence the semantics) and/or its syntax, as outlined in Table 1: (A) same semantics, same syntax (SEM_S_-SYNT_S_): a “control” version where nouns and verbs are replaced by near-synonyms (extracted using the static word embedding GloVe; Pennington et al., 2014), maintaining the active structure and the thematic role assignments (“the attorney rescued the writer”); (B) same semantics, different syntax (SEM_S_-SYNT_D_): a critical condition where the sentence is converted to passive voice while maintaining its base words and thematic role assignments (“the author was saved by the lawyer”); (C) different semantics, same syntax (SEM_D_-SYNT_S_): the other critical condition where the agent and the patient are swapped, thus changing thematic role assignments while maintaining its base words and active structure (“the author saved the lawyer”); and (D) different semantics, different syntax (SEM_D_-SYNT_D_): another “control” version where nouns and verbs are replaced with near-synonyms, the agent and patient are swapped, and the sentence is converted to passive voice, resulting in a sentence that is maximally different from the base (“the attorney was rescued by the writer”).

**Table 1.** Sample Experiment 1 materials, compared to the base sentence “The lawyer saved the author”.

	**Same Syntax (active)**	**Different Syntax (passive)**
**Same Semantics**	(A) The attorney rescued the writer	(B) The author was saved by the lawyer
**Different Semantics**	(C) The author saved the lawyer	(D) The attorney was rescued by the writer

If LMs are more sensitive to thematic roles than to structure and specific words, they should represent sentences with the same thematic role assignments (and thus similar meanings) more similarly than sentences with opposite assignments (and thus less similar meanings): the base sentence would be most similar to condition SEM_S_-SYNT_S_, followed by SEM_S_-SYNT_D_ (where thematic roles are still the same, but the syntax is different), then SEM_D_-SYNT_S_, then SEM_D_-SYNT_D_ (Note that sentence similarities might be strongly influenced by whether two sentences share the exact same nouns and verbs or, instead, use different words even if they are near synonyms; regardless of this issue, our critical comparison is between the similarity of the base to SEM_S_-SYNT_D_ and its similarity to SEM_D_-SYNT_S_). If, however, thematic roles play a less prominent role compared other sources of information in LM representations, then similarities would reflect whether sentences share structure or lexical items more strongly than they would reflect “who did what to whom”. In this case, the base sentence would be more similar to SEM_D_-SYNT_S_ than to SEM_S_-SYNT_D_. These predictions are depicted in Figure 2.

#### Language Models.

We studied sentence representations in BERT (110 M parameters; Devlin et al., 2019), GPT2-Small (117 M parameters; Radford et al., 2019), Llama 2 (7 B parameter version; Touvron et al., 2023), and Persimmon (8 B parameter version; Elsen et al., 2023) as implemented in HuggingFace. BERT and GPT2-Small have 12 layers, each with 768 hidden units and 12 attention heads. Llama2 has 32 layers, each with 4096 hidden units; and Persimmon has 36 layers each with 4096 hidden units. BERT is different from the other 3 LMs in that it (1) is bidirectional (the other LMs are unidirectional), and (2) was trained on both missing word prediction and next-sentence prediction (the other LMs were trained on next-word prediction).

#### Evaluating Representational Similarities.

For each layer in each LM, we extracted a representation of each sentence. A sentence representation consists of a distributed pattern of activity across hidden units (the residual stream). Specifically, these activities were extracted for the [CLS] token in BERT, and the ‘.’ token in GPT2, Llama2, and Persimmon (Devlin et al., 2019; May et al., 2019; Schrimpf et al., 2021). We also investigated mean pooled activations (averaging across tokens), as well as the verb token in BERT (unlike BERT, in unidirectional LMs the representations of tokens other than the final one cannot capture information about the entire sentence). The results for these analyses, reported in the Supplementary Materials, do not qualitatively differ from our main analyses.

For each sentence set, we compared the representation of the base sentence and each other condition via the cosine similarity measure (Cassani et al., 2023). Because such similarities might be influenced by a small subset of hidden units with, e.g., very high activations across all sentences (Timkey & van Schijndel, 2021), prior to computing similarities we normalized each hidden unit’s activations relative to that unit’s average and standard deviation across a large set of sentences (COCA; Davies, 2009). Cosine similarities were Fisher-transformed to render their distribution closer to Gaussian and ameliorate bias in averaging them across stimuli (Silver & Dunlap, 1987). We contrasted the four conditions in terms of their similarity to the base sentence using a non-parametric, one-way repeated-measures ANOVA (Friedman test). Significant results were followed by pairwise post-hoc, two-tailed Wilcoxon signed rank tests (Bonferroni corrected).

#### Human Judgments.

Humans can identify who did what to whom, even based on grammatical cues alone (without, e.g., plausibility information), upon careful contemplation. For example, identifying the agent and patient in “the ball was kicked by the boy” is not difficult, as common sense makes it much more plausible that the boy is the agent and the ball is the patient. But it is also possible, with some contemplation, to identify the agent and patient in sentences that do not contain such plausibility information, as “the chef was pushed by the painter”. Even though either a chef or a painter can push one another, we can rely on the syntax to identify that the painter is the agent and the chef is the patient. Still, it remains unclear how automatically and accurately we infer thematic roles based on grammatical cues alone (versus plausibility cues) and without explicit instructions to closely attend to sentence structure (Ferreira & Lowder, 2016).

To this end, we collected behavioral judgments from 120 participants, recruited via UCLA’s participant recruitment system (*n* = 3 removed due to missing responses; mean ± *SD* age: 20.19 y ± 2.72; 91 female, 25 male, 1 other). The study was approved by the university’s Institutional Review Board.

In an online experiment, participants rated pairs of sentences for their similarity, using a sliding scale between 1 (completely different) and 100 (identical). To minimize the chances that participants detect the distinctions between the conditions in Table 1 and develop an artificial strategy for solving the task, each participant made only one judgment per condition (i.e., rated the similarity between a single sentence from that condition and its corresponding base sentence). Stimuli across the four conditions came from distinct sets (no base sentence was read more than once by a participant). We created 24 experimental lists, each consisting of 4 sentence pairs and shown to 5 participants. To mask the purpose of the study, these pairs were interleaved among 5 other pairs where similarity did not require close attention to sentence structure and event roles, because it could instead be derived from general common sense (e.g., we expected two sentences about food, such as “the boy baked a cake” and “the girl devoured the sandwich”, to be more similar to one another compared to two sentences on distinct topics, such as “the soldiers stormed the camp” and “the spectators enjoyed the movie”).

We *z*-scored similarities within each participant. We then attempted to fit a linear, mixed-effects model predicting sentence similarity from condition, with random intercepts by participant and/or stimulus set: Similarity ∼ Condition + (1 | Participant) + (1 | Set). These models did not converge and showed little variance across participants (due to *z*-scoring) and across sets. Therefore, we ran a fixed-effects model predicting similarity between sentences from condition (Similarity ∼ Condition).

We emphasize that the task being done by humans here (and in Experiment 2) differs from our LM analysis (i.e., more akin to LM prompting than probing). This experiment is merely illustrative of human performance on our stimuli, confirming that the materials do indeed share meaning in the SEM_S_ conditions.

### Results

We report results for the last layer in each model, with data for all layers shown in Supplementary Figure 1. We note that semantic information is often most pronounced in middle-to-late layers, but our LMs differ in their respective number of layers; therefore, we chose the last layer for presentation in the main article because this criterion can be consistently used across LMs, unlike more ad-hoc principles based on some relative “depth”. Importantly, the critical findings hold across layers, so the results presented here are representative. Below, we use “similar” to mean “similar to one another”.

#### LMs.

**Figure 3.**
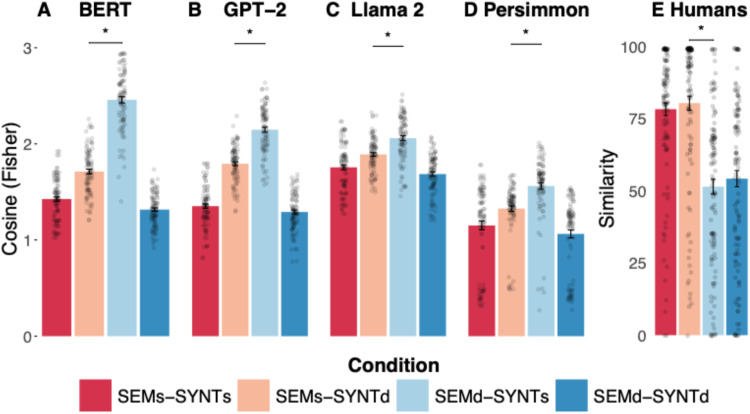
Results of Experiment 1. Similarity of the base sentences to sentences in each condition for (A) BERT, (B) GPT2, (C) Llama2, (D) Persimmon, and (E) humans. For LMs, data are shown for the last hidden layer, and cosine similarities are Fisher-transformed. Error bars show standard error of the mean; dots show individual stimulus sets (for panels A-D) or trials (for panel E).

For all four LMs, we found a significant difference between cosine similarities across conditions (Figure 3A–D, Table 2; BERT: *χ*_(3)_^2^ = 223.0, *p* < .001 Bonferroni-corrected here and below; GPT2: *χ*_(3)_^2^ = 243.17, *p* < .001; Llama2: *χ*_(3)_^2^ = 119.7, *p* < .001; Persimmon: *χ*_(3)_^2^ = 136.9, *p* < .001). Most critically, post-hoc tests revealed a surprising finding: a pair with opposite meanings (but the same syntax) was more similar than a pair with the same meaning (but different syntax; SEM_D_-SYNT_S_ > SEM_S_-SYNT_D_). This finding demonstrates that LM representations are influenced less by thematic roles and more by syntax or particular lexical items (such as the words “was” and “by” in the passive sentences).

**Table 2.** Results of Experiment 1 for each LM.^a^

**Comparison**	**BERT**	**GPT2**	**Llama2**	**Persimmon**
SEM_D_-SYNT_S_ > SEM_S_-SYNT_D_	*z* = 8.34, *p* < .001	*z* = 8.23, *p* < .001	*z* = 6.86, *p* < .001	*z* = 7.28, *p* < .001
	CI_95%_ = [0.95, 1]	CI_95%_ = [0.88, 0.98]	CI_95%_ = [0.77, 0.91]	CI_95%_ = [0.87, 0.97]
SEM_S_-SYNT_S_ > SEM_D_-SYNT_D_	*z* = 5.11, *p* < .001	*z* = 4.50, *p* < .001	*z* = 4.05, *p* < .001	*z* = 4.96, *p* < .001
	CI_95%_ = [0.66, 0.84]	CI_95%_ = [0.61, 0.79]	CI_95%_ = [0.60, 0.79]	CI_95%_ = [0.65, 0.83]
SEM_S_-SYNT_D_ > SEM_D_-SYNT_D_	*z* = 8.34, *p* < .001	*z* = 8.33, *p* < .001	*z* = 7.13, *p* < .001	*z* = 6.14, *p* < .001
	CI_95%_ = [0.89, 0.99]	CI_95%_ = [1, 1]	CI_95%_ = [0.73, 0.89]	CI_95%_ = [0.73, 0.89]
SEM_S_-SYNT_D_ > SEM_S_-SYNT_S_	*z* = 6.34, *p* < .001	*z* = 8.19, *p* < .001	*z* = 4.40, *p* < .001	*z* = 3.00, *p* = .002
	CI_95%_ = [0.71, 0.87]	CI_95%_ = [0.95, 1]	CI_95%_ = [0.57, 0.76]	CI_95%_ = [0.53, 0.73]
SEM_D_-SYNT_S_ > SEM_D_-SYNT_D_	*z* = 8.41, *p* < .001	*z* = 8.32, *p* < .001	*z* = 7.85, *p* < .001	*z* = 8.00, *p* < .001
	CI_95%_ = [1, 1]	CI_95%_ = [1, 1]	CI_95%_ = [0.85, 0.97]	CI_95%_ = [0.91, 0.99]
SEM_D_-SYNT_S_ > SEM_S_-SYNT_S_	*z* = 8.41, *p* < .001	*z* = 8.33, *p* <. 001	*z* = 7.05, *p* < .001	*z* = 7.45, *p* < .001
	CI_95%_ = [0.97, 1]	CI_95%_ = [1, 1]	CI_95%_ = [0.76, 0.9]	CI_95%_ = [0.85, 0.97]

^a^ 95% confidence intervals show the probability that, for a randomly sampled stimulus set, the difference denoted on the left-most column would be observed in the right direction. Intervals were estimated using 5000 bootstrap samples.

For all LMs, post-hoc tests also revealed that (1) the two control conditions differed from one another as expected (SEM_S_-SYNT_S_ > SEM_D_-SYNT_D_); (2) a pair with different syntax but shared meaning was more similar than the maximally different control pair (SEM_S_-SYNT_D_ > SEM_D_-SYNT_D_) but was also more similar than the maximally similar control pair (SEM_S_-SYNT_D_ > SEM_S_-SYNT_S_). The latter effect perhaps reflects the fact that SEM_S_-SYNT_D_ sentences shared the same nouns and verbs with the base sentence, whereas SEM_S_-SYNT_S_ instead consisted of near synonyms; and (3) a pair with the same syntax but different meaning was more similar than the maximally different pair (SEM_D_-SYNT_S_ > SEM_D_-SYNT_D_), but also more similar than the maximally similar pair (SEM_D_-SYNT_S_ > SEM_S_-SYNT_S_). Again, this latter effect perhaps reflects lexical overlap between pairs of sentences.

#### Human Judgments.

A fixed-effects model predicting similarity between sentences from condition had an adjusted *R*^2^ of 0.25, *F*_(4,458)_ = 40.01, *p* < .001 (Figure 3E). Most critically, post-hoc tests revealed that a pair with the same meaning (but different syntax) was more similar than a pair with opposite meanings (but the same syntax; SEM_S_-SYNT_D_ > SEM_D_-SYNT_S_, *t*_(458)_ = 9.27, *p* < .001). This pattern is the opposite of what was found for LMs, and demonstrates that thematic roles exert a stronger influence on human similarity judgments than syntax does.

Post-hoc tests also found that: (1) the two control conditions differed from one another in the expected direction (*t*_(458)_ = 7.83, *p* < .001); (2) a pair with the same meaning but different syntax was more similar than the maximally different control pair (SEM_S_-SYNT_D_ > SEM_D_-SYNT_D_, *t*_(458)_ = 8.13, *p* < .001), but did not differ from the maximally similar control pair (SEM_S_-SYNT_D_ vs. SEM_S_-SYNT_S_, *t*_(458)_ = 0.29, *p* = 1); and (3) unlike in LMs, but consistent with a strong influence of thematic roles, a pair with different meanings but shared syntax was less similar than the maximally similar pair (SEM_D_-SYNT_S_ < SEM_S_-SYNT_S_, *t*_(458)_ = 8.96, *p* < .001); and, despite sharing syntax, this pair did not differ from the maximally different pair (SEM_D_-SYNT_S_ vs. SEM_D_-SYNT_D_, *t*_(458)_ = 1.15, *p* = 1).

We do not explicitly compare human and LM data, because the former are based on explicit ratings in response to prompts whereas the latter quantify internal representations (although such comparisons have been carried out in previous studies comparing LMs and humans, e.g., Hu et al., 2024; Iaia et al., 2025; Kodali et al., 2025; Lau et al., 2020; Lu et al., 2024; they are also common in studies of visual processing, e.g., Hebart et al., 2020; Jozwik et al., 2017; Mahner et al., 2025; Muttenthaler et al., 2023; Takahashi et al., 2025; Tarigopula et al., 2023). Nonetheless, the behavioral data provide some validation of our stimuli, and demonstrate that thematic role information is psychologically salient for human comprehenders even in the absence of plausibility cues and task demands.

## EXPERIMENT 2

**Figure 4.**
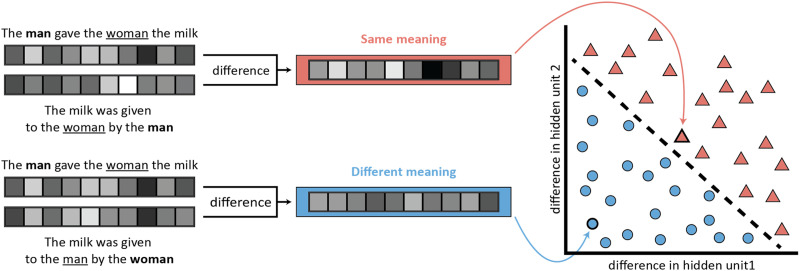
Basic approach of Experiment 2. We test whether a linear classifier (SVM) can find a subspace which separates pairs of sentences with the same meaning (left, top) from pairs of sentences with different meanings (left, bottom). In each sentence, the agent is bolded and the (proto-)patient is underlined (here, the latter is a recipient). To train the SVM, we first extract sentence embeddings from a given layer of a LM and then subtract the embeddings of pairs of sentences to obtain a difference vector (middle). The illustrated example on the right shows the distribution of difference vectors for two hidden units, including sentence pairs with the same (red triangles) or different (blue circles) meanings. Even though the first unit (*x*-axis) cannot distinguish difference vectors that represent same- vs. different-meaning sentences, and the second unit (*y-*axis) similarly cannot distinguish them, there is a subspace where the two classes of difference vectors are linearly separable and fall on opposite sides of a “boundary” (dashed line).

A cognitively plausible representation of sentences should feature thematic roles as a main component (Rissman & Majid, 2019; Ziegler & Snedeker, 2018); for this reason, Experiment 1 quantified LM representations as distributed activity patterns across all hidden units in a given layer. However, thematic roles might instead be encoded by a low-dimensional subspace (for instance, a small subset of units, with other units representing unrelated information, e.g., syntax or lexical semantics). Thus, even if LMs do not emphasize thematic roles—as evidenced in Experiment 1—perhaps they still extract this information, i.e., they have the capacity for representing this information, like humans do (Figure 4).

Experiment 2 thus addressed two questions. First, we asked: are thematic roles robustly represented anywhere within the residual stream, even in some subspace? If this were the case, the analyses of Experiment 1 may not have been able to discover this more “localized” representation. To evaluate this possibility, we again used pairs of sentences that either shared the same agent and the same patient or had reversed thematic role assignments. We first extracted activity patterns across all hidden units for each sentence, and then submitted paired representations to an algorithm that tried to find any information, in any subspace, that could classify whether that pair of sentences shared common thematic role assignments or not. In other words, whereas Experiment 1 examined the relative prominence of thematic roles vs. syntactic structure, Experiment 2 served as a more direct test of whether LMs encode thematic role information.

Our second question was whether thematic role information was available in components of LMs other than hidden units, namely, in the attention heads. Attention heads are a computational mechanism that influences how the representation of each word (or, more generally, token) is transformed from one layer of hidden units to the next. Specifically, each head determines how words in a sentence are related to one another; these relations reflect various sources of information, which are inferred automatically during training, and might include semantics, syntax, linear position, etc. To this end, an attention head assigns numerical weights between words (i.e., specifying how much a word should “attend” to another): in bidirectional transformers (like BERT), each word attends to every other word and to itself, whereas in unidirectional transformers (like GPT2, Llama2, and Persimmon), each word attends only to itself and to previous words (i.e., it cannot “look ahead”). The representation of a given word is then updated to incorporate information from all the words it attends to, with attention weights determining the relative contribution of each word to the resulting representation (though the exact contribution of each word also depends on the information it carries). Different heads assign different weights, and extract different aspects of information from the words they attend to. These differences produce distinct representations for a word, which are combined across all heads in a given layer and further transformed before being added to that word’s representation from the previous layer (in the residual stream) and passed on to the next set of hidden units. To test whether any attention heads capture thematic role information, for each head we extracted attention weights between content words in a sentence, and used the same classification procedure described above on pairs of sentences that had either shared or reversed thematic role assignments.

### Methods

#### Stimuli.

An algorithm that attempts to classify whether or not two sentences share the same thematic role assignments, based on any information in the hidden units of an LM, can solve the task based on simple “tricks”. For example, if some units represent the position of each noun in the sentence, and some units represent the existence of the word “by”, then two sentences have the same meaning if the nouns are in opposite orders across the two sentences, and only one has the word “by”. To prevent the use of such heuristics, in Experiment 2 we used a more diverse and challenging stimulus set. It consisted of ditransitive sentences with a variety of structures (e.g., “the man gave the milk to the woman”, “it was the woman that was given the milk by the man”), where no global “trick” can be applied to infer whether sentences shared thematic role assignments or not. A single stimulus set used 12 structures, each with two versions having opposite agent-patient assignments, for a total of 24 sentences. Structures varied in whether they were active or passive, used a double- or prepositional-object construction (DO vs. PO), and were in simple or cleft form (with a subject, direct object, or indirect object focus). We generated 50 sets of such 24 sentences, for a total of 1,200 sentences. Example stimuli are provided in Table 3.

**Table 3.** Example stimuli for Experiment 2.^a^

**Active / Passive**	**DO / PO**	**Simple / cleft**	**Sentence**
Active	DO	Simple	**The man** gave the woman the milk.
		Cleft (subject)	It was **the man** who gave the woman the milk.
		Cleft (dir. object)	It was the milk that **the man** gave the woman.
	PO	Simple	**The man** gave the milk to the woman.
		Cleft (subject)	It was **the man** who gave the milk to the woman.
		Cleft (dir. object)	It was the milk that **the man** gave to the woman.
		Cleft (indir. object)	It was the woman who **the man** gave the milk to.
Passive	DO	Simple	The woman was given the milk by **the man**.
		Cleft (indir. object)	It was the woman who was given the milk by **the man**.
	PO	Simple	The milk was given to the woman by **the man**.
		Cleft (dir. object)	It was the milk that was given to the woman by **the man**.
		Cleft (indir. object)	It was the woman who the milk was given to by **the man**.

^a^ The agent in all sentences is shown in bold (the man). Each stimulus set consisted of 12 more sentences (one more version of each structure) with the opposite thematic role assignment (in this example, woman as agent). Abbreviations: dir = direct; indir = indirect.

These stimuli also remedy one potential issue with the stimuli from Experiment 1. In that experiment, sentences that shared thematic role assignments always differed in their lexical items: one sentence was active (“the lawyer saved the author”) and the other was passive (“the author was saved by the lawyer”), so only the latter included the words “was” and “by”. In contrast, sentences that had opposite thematic role assignments (“the lawyer saved the author” vs. “the author saved the lawyer”) had complete lexical overlap. Our results of Experiment 1 could therefore indicate a strong effect of lexical overlap, where sentence pairs with the same (vs. reversed) thematic role assignments have less similar representations because of lower overlap. This explanation would still support our conclusion that thematic role information exerts only a weak influence on overall representational similarities in LMs (a system that robustly captures such information should not be modulated so dramatically by a couple of closed-class words). However, we wanted to test LMs with stimuli where thematic role alignment and lexical overlap did not consistently pull in opposite directions. In Experiment 2, sentence pairs with the same thematic role assignments could have either more overlap, less overlap, or an equal amount of overlap compared to pairs with opposite assignments (see Table 3).

#### LMs: Hidden Units.

Prior to training a classifier algorithm on pairs of sentences, we performed the same analysis as in Experiment 1, computing cosine similarities for every pair of sentences within each stimulus set based on the pattern of activity distributed across all hidden units in each layer. We tested whether similarities for pairs that shared thematic role assignments were higher than for pairs with opposite assignments, splitting the analysis by whether the respective structures of the two sentences in a pair differed in 0, 1, 2, or 3 of the syntactic features described above. An example pair of sentences with 0 changes and the same thematic role assignments: “it was the man who gave the woman the milk” and “it was the milk that the man gave the woman”; both are active, use the direct object construction, and have a cleft, but each cleft has a different focus. A pair of sentences that differ in 1 feature are “the man gave the woman the milk” and “it was the man who gave the woman the milk”, as they are both active and DO, but the second sentence has a cleft. A pair of sentences that differ in 2 features are “the man gave the woman the milk” and “the milk was given to the woman by the man”, as the second sentence is passive and has a cleft. A pair of sentences that differ in 3 features are “the man gave the woman the milk” and “it was the milk that was given to the woman by the man”, as the second sentence is passive, has a cleft, and a prepositional object construction. There are other ways to split our sentence pairs based on their syntactic differences, and we do not claim that our distinctions are more cognitively relevant; we chose this scheme because it reflects how we constructed the stimuli. We emphasize that this analysis is exploratory in nature. *p*-values were Bonferroni-corrected for multiple comparisons across layers, separately for each LM.

For our main analysis, for each layer in each LM, we trained a support vector machine (SVM) algorithm to distinguish between “same meaning” vs. “different meaning” sentence pairs (i.e., pairs with matching vs. opposite thematic role assignments; see also our Supplementary Materials for a logistic regression analysis with similar results). We use a linear classifier, consistent with previous research that has found conceptual information in hidden layers with this approach (Lee et al., 2024; Nanda et al., 2023; Ramezani et al., 2025; Seyffarth et al., 2021; also see Discussion). To this end, for each pair of sentences, their respective distributed representations were subtracted and the absolute value of the difference was submitted as a training instance to the algorithm (we also attempted to train the algorithm on the concatenated representations of two sentences in a pair; results were similar or weaker). In a 66-fold cross-validation, a separate SVM was trained on each possible subset of 10 of the 12 sentence structures and tested on the remaining two held-out structures.

Specifically, for each fold, training data were generated from 1,000 sentences: 20 out of the 24 sentences in each stimulus set (10 structures × 2 possible agent-patient assignments). Within each of our 50 stimulus sets, we used nearly all possible sentence pairs, excluding pairs consisting of two versions of the exact same structure (e.g., passive + prepositional object + cleft with a direct object focus), because those pairs always had opposite thematic role assignments. Overall, each fold included 9,000 training pairs. Because the held-out test data included 2 structures, each with two versions (differing in thematic role assignments), there were 4 test pairs per stimulus set, for a total of 200 pairs per fold (again, excluding pairs consisting of two versions of the same structure).

For each layer of each LM, binary classification accuracy was tested against chance performance (0.5) using a one-sample *t*-test. *p*-values were Bonferroni-corrected for multiple comparisons across layers, separately within each LM.

This test is very strict, as classifiers need to generalize to out-of-distribution structures. It is possible to conduct a less strict analysis where the training and testing both include examples of all 12 sentences templates. We also conducted this easier analysis, training classifiers on sentence pairs including all structures from 49 of these sets, and tested on the remaining, held out set. In this set up, generalization is required across lexical items, but not across syntactic structures. The overall pattern of results was similar, although accuracies did increase. We report these findings in our Supplementary Materials. We believe that performance on this easier setup may be driven to some extent by superficial strategies (e.g., memorizing the templates) and therefore focus on the stricter analysis.

#### LMs: Attention Heads.

For each attention head in each layer, we studied attention patterns assigned between content words in each sentence. Namely, we extracted each sentence’s attention weights between every pair of the following words: subject (which, depending on sentence structure, was the agent or patient), indirect object (patient or agent), verb, and direct object; we excluded attention from the verb to the direct object and vice versa because these involved neither agent nor patient. Using these vectors of 10 attention weights per sentence, the same SVM analyses described above were conducted to classify sentence pairs with matching vs. opposite thematic role assignments. However, here we used a concatenation of the two vectors of a pair of sentences rather than their difference, because this representation resulted in better performance and we wanted to give LMs the best opportunity to succeed. Because the relative position of words varied across sentence structures, this analysis sometimes necessitated “forward-looking” attention (i.e., from a previous word to a future word). However, attention in most of our models is only backward-looking (unidirectional), so our analysis was limited to BERT, which has bidirectional attention. *p*-values were Bonferroni-corrected for multiple comparisons across all attention heads and layers (12 heads per layer × 12 layers = 144 tests).

#### Human Judgments.

We recruited two samples (121 and 120 participants, respectively) through UCLA’s participant recruitment system for an online experiment. Participants rated pairs of sentences for their similarity as in Experiment 1. In the first sample, each participant judged only two critical pairs of sentences: in one pair, the two sentences shared the same thematic role assignments, and in the other they had opposite assignments. These two trials were from different stimulus sets. Due to a coding error, in this first sample, we only sampled 12 out of the 50 stimulus sets. Furthermore, due to another error, each participant rated one pair in which the two sentences differed in 3 syntactic features (passive-DO-simple vs. active-PO-cleft), and another pair in which they differed in 2 syntactic features (active-simple vs. passive-cleft, both with a PO). Five filler trials were included as in Experiment 1.

The second sample of participants rated four critical pairs of sentences alongside 18 filler trials, and the full stimulus set was sampled across participants. In two pairs—one with shared and one with opposite assignments—the two sentences differed in a single syntactic feature (for some participants, passive-simple with DO vs. PO; for other participants, active with simple vs. cleft form, either both with a DO or both with a PO). In the two remaining pairs—again, one with shared and one with opposite assignments—the two sentences differed in two syntactic features (for some participants, passive-simple vs. active-cleft, both with a PO; for other participants, active-DO vs. passive-PO, both in simple form).

We *z*-scored similarities within each participant. We then tested whether similarities for sentence pairs that shared thematic role assignments were higher than for pairs with opposite assignments, splitting the analysis by whether the respective structures of the two sentences in a pair differed in 1, 2, or 3 of the syntactic features. For pairs differing in one feature, we fitted a linear, mixed-effects model predicting sentence similarity from condition (shared vs. opposite thematic roles), with random intercepts by participant and by stimulus set: Similarity ∼ Condition + (1 | Participant) + (1 | Set). For pairs differing in two features, we fitted a similar model but without a random intercept by participant, because some participants (namely, those from the first sample) only saw a single sentence pair of this format. For pairs differing in three syntactic features, we did not include a random intercept by participant for the same reason; the model including a random intercept by stimulus set did not converge, so it was reduced to a fixed effects model predicting similarity rating from condition. *p*-values were Bonferroni-corrected across these 3 models.

### Results

#### LMs: Sentence Similarity.

**Figure 5.**
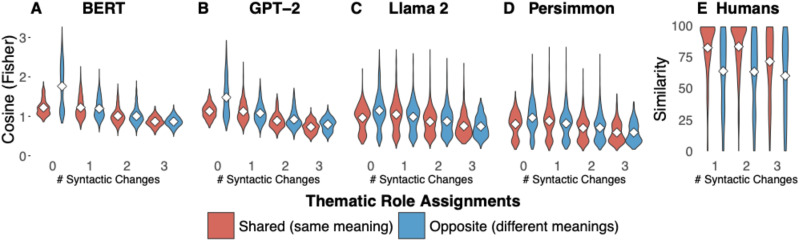
Results of Experiment 2, showing similarity patterns between sentence pairs that have shared (red) or opposite (blue) thematic role assignments, for (A) BERT, (B) GPT2, (C) Llama2, (D) Persimmon, and (E) humans. For LMs, data are shown for the last hidden layer, and cosine similarities are Fisher-transformed. Violin plots show the distribution over sentence pairs. White diamonds show the mean.

When considering sentence representations distributed across all hidden units within a given layer, we did not find evidence of robust representation of thematic roles (Figure 5). Only a minority of cases showed the expected pattern, whereby pairs of sentences that share thematic role assignments are more similar than pairs with opposite assignments. For all LMs, we also observed a strong influence of syntax on sentence similarity: sentence pairs differing in one syntactic feature were overall more similar than pairs differing in two syntactic features, which were overall more similar than pairs differing in three syntactic features; this pattern held regardless of whether pairs shared the same thematic role assignments or had opposite assignments.

In BERT, for sentence pairs differing in one, two, or three syntactic features, similarity did not vary as a function of whether the pair had the same thematic role assignments (one feature: *z* = 1.94, *p* = .21, Bonferroni-corrected here and below; two features: *z* = .52, *p* = 1; three features: *z* = .0077, *p* = 1; Figure 5A). Pairs with no differences in syntactic features showed a pattern that is opposite of what would be expected from a representation that is sensitive to thematic roles: pairs with opposite thematic role assignments were more similar than pairs which shared thematic role assignments (*z* = 17.05, *p* < .001).

In GPT2, for sentence pairs differing in one syntactic feature, pairs which shared thematic role assignments were more similar than pairs with opposite assignments, consistent with sensitivity to thematic roles (*z* = 5.06, *p* < .001; Figure 5B). However, pairs with no differences in syntactic features, as well as pairs differing in 2 or 3 features, showed the opposite pattern (no difference: *z* = 11.05, *p* < .001; two features: *z* = 2.78, *p* = .02; three features: *z* = 4.46, *p* < .001).

In Llama2, for sentence pairs differing in one syntactic feature, pairs which shared thematic role assignments were more similar than pairs with opposite assignments, consistent with sensitivity to thematic roles (*z* = 3.52, *p* = .001; Figure 5C). However, pairs with no differences in syntactic features showed the opposite pattern (*z* = 6.69, *p* < .001). For pairs that differed by 2 or 3 features, similarity did not vary as a function of whether the pair had the same meaning (two features: *z* = .71, *p* = 1; three features: *z* = .15, *p* = 1).

Finally, the results for Persimmon mirrored those of Llama2. For sentence pairs differing in one syntactic feature, pairs which shared thematic role assignments were more similar than pairs with opposite assignments, consistent with sensitivity to thematic roles (*z* = 4.70, *p* < .001; Figure 5D). However, pairs with no differences in syntactic features showed the opposite pattern (*z* = 6.58, *p* = .001). For pairs that differed by 2 or 3 features, similarity did not vary as a function of whether the pair had the same meaning (two features: *z* = .20, *p* = 1; three features: *z* = .52, *p* = 1).

#### Human Judgments.

Human similarity judgments showed higher sensitivity to thematic role information compared to LMs (Figure 5E): they rated sentence pairs which shared thematic role assignments as more similar than pairs with opposite assignments, regardless of whether the two sentences in a pair differed in a single syntactic feature (*z* = 6.25, *p* < .001), in two features (*z* = 7.90, *p* < .001), or in three features (*t*_(118)_ = 3.04, *p* = 0.004; this test was between- rather than within-participants). For pairs differing in either one or two syntactic features, 70.3% of participants provided ratings in this direction (CI_95%_ = [62%, 78.5%], based on 5,000 bootstrap samples; such a percentage could not be calculated for pairs differing in 3 syntactic features, because no participant saw more than one sentence pair of this format; see Methods).

#### SVM: Hidden Units.

**Figure 6.**
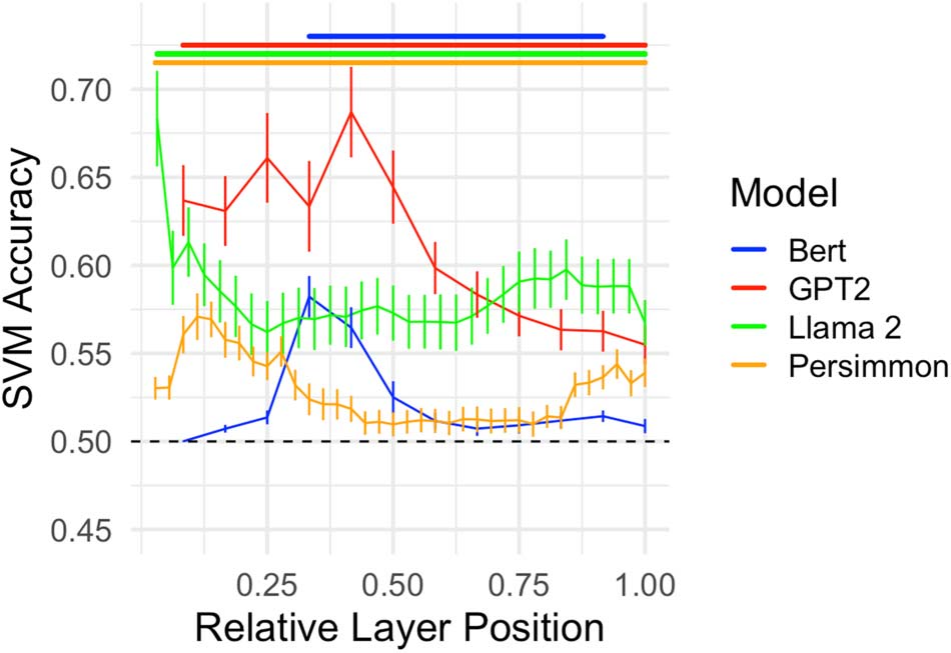
Classification accuracies for SVMs, trained on distributed activations across hidden units to predict whether two sentences had shared vs. opposite thematic role assignments (i.e., “same” vs. “different” meanings). A separate SVM was trained per layer, and the *x*-axis shows relative layer position. Different curves correspond to different LMs. Error bars show standard errors of the mean. A black, dotted line denotes chance classification. Colored, horizontal lines represent where classification accuracy is significantly above chance.

Classification accuracies of sentence pairs with shared vs. opposite thematic role assignments (i.e., “same” vs. “different” meanings), for each layer in each LM, are shown in Figure 6. Many SVM results significantly exceeded chance level (0.5), but with a relatively small effect size, mostly below 0.6 accuracy. The highest classification accuracy was obtained for GPT2, layer 5. For this layer, accuracy exhibited high variance, ranging from 100% accuracy to 33% accuracy across folds, with the latter suggesting overfitting; and 36% of the folds showed chance accuracy or lower (layer 1 of Llama2 also demonstrated relatively high accuracy, and similarly exhibited high variance in accuracy across folds).

Even though we do not directly compare LM data to human performance (see Discussion), we note that SVM performance in this GPT2 layer was descriptively similar to human ratings. As a reminder, human similarity ratings were significantly higher for sentence pairs with matching vs. opposite thematic roles; for sentence pairs differing in either one or two syntactic features, 70.3% of participants provided similarity ratings in this direction, i.e., human implicit “classification accuracy” was 0.703. In layer 5 of GPT2, the 95% confidence interval of classification accuracy was [0.636, 0.738] (5,000 bootstrap samples across folds).

The overall low classification accuracy across models and layers might, in part, reflect the difficulty of processing sentences with cleft structures, which constitute two thirds of our stimuli. Indeed, classification accuracy for held-out sentences that included clefts tended to be lower than accuracy on held-out sentences that did not include those structures. However, we note that previous work has demonstrated that even our smaller LMs (i.e., BERT and GPT2) are able to process clefted sentences, as evidenced in their next-word predictions (Da Costa & Chaves, 2020; see also Hu et al., 2020; Warstadt & Bowman, 2020).

#### SVM: Attention Heads.

**Figure 7.**
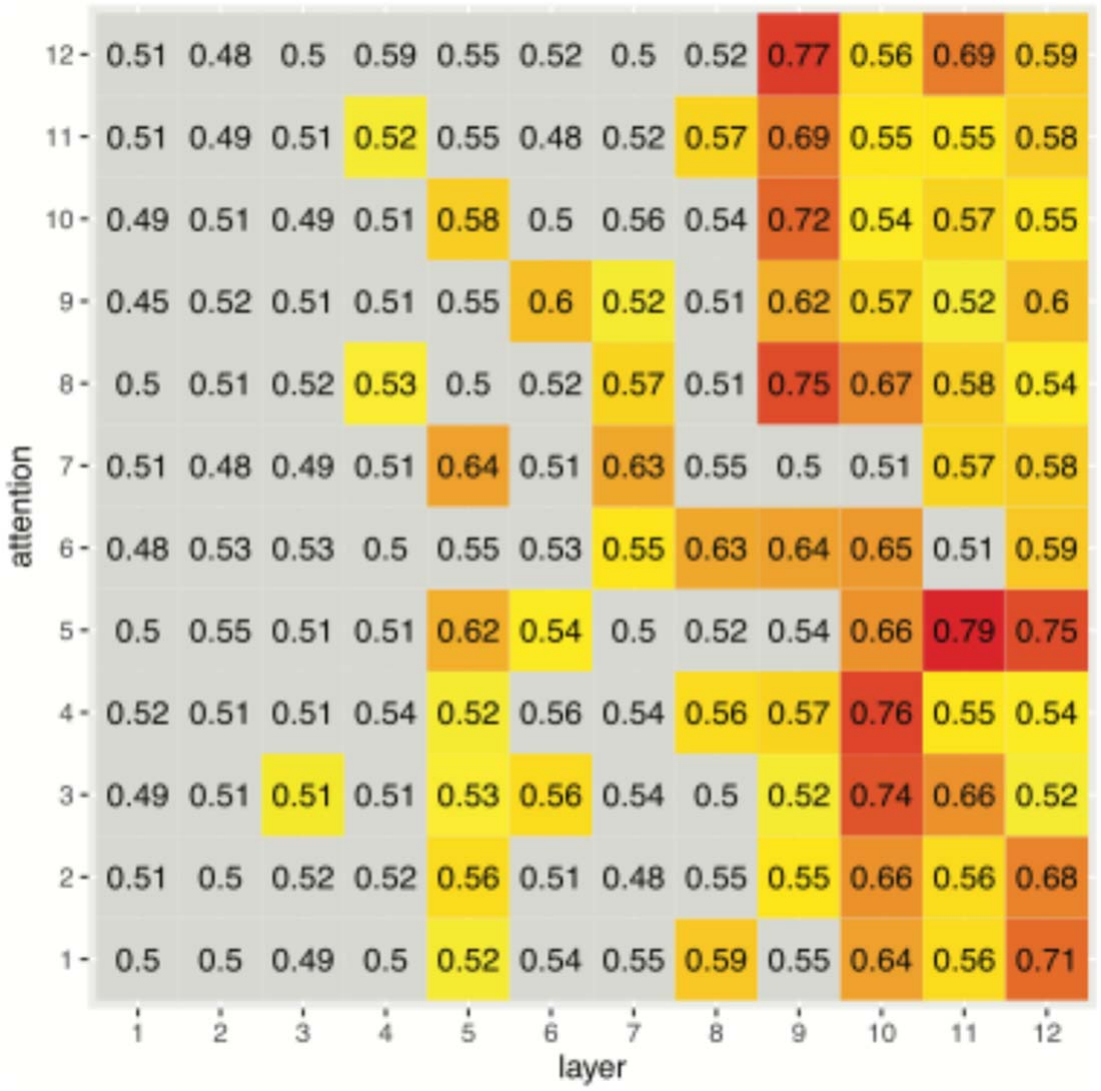
Classification accuracies for SVMs, trained on patterns of attention in BERT to predict whether two sentences had shared vs. opposite thematic role assignments (i.e., “same” vs. “different” meanings). A separate SVM was trained per attention head, and each cell in the matrix shows the result for one head (row) in one layer (column). Accuracies significantly above chance are colored (Bonferroni-corrected for multiple comparisons across all heads).

Classification accuracies of sentence pairs with shared vs. opposite thematic role assignments (i.e., “same” vs. “different” meanings), for each attention head in each layer of BERT, are shown in Figure 7. Several heads had high accuracy, and we highlight head 5 in layer 11, which had the highest accuracy: 0.79. SVM performance, broken down by the number of syntactic features that differed between sentences in a pair, was as follows: 0 features, 0.801; one feature, 0.794; two features, 0.792; and three features, 0.784. The 95% confidence interval of classification accuracy across folds was [0.784, 0.801] (based on 5,000 bootstrap samples), compared to our proxy of human implicit “classification accuracy” of 0.703.

**Figure 8.**
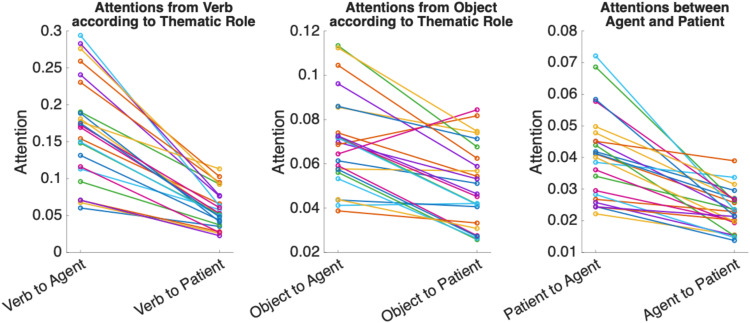
Attention patterns between different thematic roles, for BERT’s head 5 in layer 11. Each line corresponds to one of 24 sentence types (12 structures × thematic role assignments), averaged across stimulus sets.

To characterize this head’s function (Figure 8), we contrasted its attention patterns (1) from the verb to the agent vs. patient; (2) from the direct object to the agent vs. patient; and (3) from agent to the patient vs. vice versa. Each comparison was carried out in a linear, mixed-effects model. Attention weights, which are restricted between [0,1], were logit-transformed (this did not change the results) and modeled with a fixed effect of direction (towards the agent vs. patient) and random intercepts and slopes by stimulus set and by structure: Attention ∼ Direction + (1 + Direction | Set) + (1 + Direction | Structure; based on the 12 structures in Table 3). The verb (*t*_(51.48)_ = 13.76, *p* < .001) and direct object (*t*_(36.48)_ = 5.18, *p* < .001) both allocated more attention to the agent than to the patient, and patients directed more attention to agents than vice versa (*t*_(35.60)_ = 7.76, *p* < .001). These patterns held across most sentence structures regardless of the grammatical positions of agent and patient. Therefore, they robustly reflect thematic roles, independent of syntax.

## DISCUSSION

This study asked whether Language Models (LMs) understand sentences in the minimal sense of representing “who did what to whom”. In Experiment 1, we found that the overall geometry of LM distributed activity patterns failed to capture this information: similarities between sentences did not reflect whether they shared thematic role assignments. Human judgments, in contrast, were strongly driven by this aspect of meaning. In Experiment 2, we found limited evidence that thematic role information may be even represented more narrowly, in a subspace of the residual stream. Within the high-dimensional space of activity patterns in hidden units, we could find subspaces that often allowed for significant classification of whether sentence pairs had shared vs. opposite thematic role assignments, but the effect sizes were small, with few exceptions. Even in the best-performing case, which appeared descriptively comparable to human performance, the representation of thematic roles did not seem robust across syntactic structures. However, thematic role information was more reliably available in a large number of attention heads, demonstrating LMs have the capacity to extract thematic role information (and, in some cases, information present in attention heads descriptively exceeded human performance).

These results provide an important characterization of the ability of LMs to understand language: thematic roles are tightly linked to understanding linguistic input as such, in contrast to aspects of comprehension such as common-sense reasoning or logical inference which reflect non-linguistic aspects of thinking (Mahowald et al., 2024). Even in our relatively simple semantic task of mapping sentence structure onto thematic roles, LMs do not give meaning a prominent role—at least not in their hidden units—despite possessing the ability to extract this information. If we take the distributed patterns of activity across hidden units to be a representation of a sentence, as is often done in studies comparing LMs to human data (Antonello et al., 2021; Caucheteux & King, 2022; Schrimpf et al., 2021; Tang et al., 2023; Tuckute et al., 2024), then our findings suggest that training LMs on word prediction is sufficient for learning what thematic roles are, but perhaps not for representing them in a human-like way.

As stated in the introduction, LMs can perform thematic role assignment if they are directly fine-tuned on this task. However, our study asked whether robust representations of thematic roles could result from training on word prediction exclusively, in order to understand how a general linguistic objective affects LM semantic capabilities. Our work thus adds to the existing endeavor of characterizing models that lack fine-tuning (for a review, see: Chang & Bergen, 2024). Probing such models is crucial as they are the ones that are commonly compared to human behavior and brain activity. For instance, one important interpretation of our findings is that whatever brain activity can be predicted from the hidden activations of LMs, it likely does not reflect thematic roles (because such information is not reliably represented in hidden activations).

Can we interpret the relationship between the (pre-)training objective of LMs and thematic role assignment with reference to theories of human language acquisition? Apart from strongly nativist theories which claim that infants have innate linguistic knowledge of possible mappings between syntactic positions and thematic roles (e.g., Pinker, 2009), most contemporary theories assume that this mapping is learned from linguistic input via some form of distributional learning. One instantiation of a distributional learning mechanism is prediction of upcoming linguistic input, which is not only the basis of LM pre-training but also a fundamental process in the human mind (e.g., Ryskin & Nieuwland, 2023).

However, humans and LMs are critically different in their starting state. Most acquisition theories posit that infants start out with abstract, non-linguistic knowledge of concepts like “agent” and “patient”, which is either innate or quickly learned pre-linguistically (Rissman & Majid, 2019), and which is used to construct mental representations of events in the world. This knowledge supports and constrains the acquisition of syntax and of the mapping rules between syntax and semantics. For example, infants observe events, mentally represent them, and infer how the linguistic strings that they are exposed to relate to those conceptual representations (theories disagree on what additional types of knowledge constitute the starting state, and on how learning proceeds; for a review and a recent proposal, see Hartshorne & Snedeker, 2026). In contrast to humans, LMs do not have preexisting concepts; in fact, their learning trajectory is opposite to that of humans: they begin from forms, not from meanings.

In this sense, the LMs we study are not models of acquisition. Nonetheless, one can still ask if they are good models of the adult state of the linguistic mind—the question that guides studies comparing LMs to behavioral and neural data. Could LMs develop the same linguistic abilities that humans have (or, at least, a non-trivial subset of them), but get there through a different route? This question is intriguing given the unexpected power of distributional learning (Contreras Kallens et al., 2023; Piantadosi, 2023). Even prior to LMs, models of contextless word meanings (“static” word embeddings) have demonstrated how much (lexical) semantic knowledge is implicitly embedded in linguistic strings (e.g., Grand et al., 2022; Liu et al., 2025; Utsumi, 2020). LMs then showed that a large amount of syntactic information can be learned from word prediction alone. How far can domain-general distributional learning go, in terms of providing a system with human-like linguistic abilities? A critical component in this research program moves from sentence structure to sentence meaning, and our study belongs to this burgeoning line of work.

Our findings are consistent with the broader claim that the success of LMs in syntactic processing does not guarantee similar success in semantic processing (Weissweiler et al., 2022). Indeed, despite the impressive syntactic capabilities of LMs (e.g., Manning et al., 2020; McCoy et al., 2023; Wilcox et al., 2021, for reviews, see: Chang & Bergen, 2024; Futrell & Mahowald, 2026; Linzen & Baroni, 2021), prior work has demonstrated that LMs trained on word prediction alone have limited understanding (though this has often been evaluated on non-linguistic aspects of understanding): LMs struggle with tracking the state of entities in a text (Kim & Schuster, 2023), sometimes refer to entities that do not exist (Schuster & Linzen, 2022), and make predictions that are only weakly sensitive to event roles (Ettinger, 2020).

At the same time, this study adds to a growing literature suggesting that attention heads—a critical component of LMs that is distinct from hidden units—contain important linguistic information. Previous work has found that attention heads and circuits of attention can identify parts of speech and syntactic dependencies (Clark et al., 2019; Manning et al., 2020; Wang et al., 2022) and, moreover, that those computations are influenced by semantic information (McGee et al., 2026). Here, we demonstrate that thematic role information is also reliably captured by attention heads. The relationship between heads that identify syntactic relations and those that identify semantic relations remains an open question for future studies. Other work, focusing on BERT’s attention heads, has shown that they tend to be “overparametrized”, with many heads attending to the same information (e.g., noun-pronoun or verb-subject links), and that disabling some heads can even improve performance (Kovaleva et al., 2019). Our findings are consistent with these claims, as we found many attention heads that appear to be sensitive to thematic roles. Whereas our attention head analysis focused on BERT, because our evaluation method required a model with bidirectional attention, future work may test whether heads in unidirectional models also extract thematic role information. Future work may also consider the causal influence of thematic role information in attention heads (or circuits of attention; Wang et al., 2022) on next word prediction and LM behavior more broadly.

The fact that certain attention heads in BERT have learned to reliably identify thematic roles across a variety of syntactic structures suggests that thematic roles were useful for solving the model’s training objective—predicting a missing word in a sequence (i.e., masked language modeling). In other words, identifying “who did what to whom” may help the model to disambiguate or predict masked words, such as verbs that depend on roles (e.g., “the waiter [MISSING] the patron” might require verbs like “served”, but “the patron [MISSING] the waiter” might require verbs like “tipped”). Yet, despite this clear functional role at the level of attention, thematic role information appears to be “washed out” or transformed in the hidden activations (i.e., the residual stream), where sentence representations reflect lexical items and the syntax connecting them, but do not consistently capture event structure. What might account for this discrepancy between attention heads and hidden activations?

One possibility is that thematic role information is used locally: during training, attention heads isolate the agent and patient to adjust the representation around the missing word (i.e., the prediction site), but this information is then folded into a more abstract representation as it flows through the layers. In this sense, thematic role information may act like temporary scaffolding: essential for constructing a prediction-relevant context, but not part of the final structure that persists in the hidden activations. Importantly, in this case thematic information is not discarded in a functional sense. Even if we could not easily decode thematic roles from the hidden activations, these roles may still exert a causal influence on an LM’s output. The identity of the agent and patient may modulate the hidden activations in subtle ways that influence lexical choice (e.g., helping the model choose between “served” vs. “tipped”), without encoding the agent or patient explicitly anymore. Put differently, once the model has used attention to determine that “the waiter” is likely the agent, this information might shift activation patterns in a way that biases the prediction of, e.g., missing verbs towards particular meanings, without needing to retain discrete “agent” and “patient” tags. However, this possibility is unlikely, because the hidden representations of two sentences with the same thematic role assignments should still shift in more similar ways than the representations of two sentences with reversed assignments. This pattern was not observed in our analyses of hidden activations. Still, it would be informative to test whether, and in what ways, LM outputs are sensitive to thematic roles (in the absence of plausibility cues) by testing their next-word predictions on controlled experimental stimuli.

A second, related possibility is that even though thematic role information in attention heads is abstract—i.e., an agent role is detected regardless of the nouns and verbs in the sentence—this information modulates hidden activations in a more lexically-specific way (e.g., Diego-Simón et al., 2026). If LMs manipulate representations in the service of next-word prediction, and such predictions heavily depend on fine-grained meaning, then hidden activations may distinguish between fine-grained, or verb-specific, roles: they may reliably distinguish between the agent vs. patient of a pulling action (the “puller” vs. the “pulled”), but this distinction would not share representational resources with another reliable distinction, e.g., between the agent vs. patient of a rescuing action (“rescuer” vs. “rescued”). Such verb-specific distinctions between agent and patient could not be detected by our SVM analysis, which required generalization across verbs (for discussions about the relationship between item-specific and abstract linguistic knowledge in humans, see Ambridge, 2020a, 2020b; Tomasello, 1992, 2003).

A third possibility is that, in an LM’s training corpus, thematic role information interacts with other constraints that support word prediction, such as plausibility cues and world knowledge. Previous work has indeed demonstrated that LMs can distinguish plausible vs. implausible events (Kauf et al., 2023). When such constraints are missing—as in our controlled stimuli—thematic role information is no longer strongly represented in the hidden activations. In other words, the interaction between multiple constraints is necessary to “elevate” thematic role information and allow it to play a prominent role in the hidden activations.

A fourth possibility is that thematic role information is coded in the hidden layers in a non-linear way, such that our linear probe could not identify it. That is, we used a specific similarity metric (cosine) and rather simple classifiers (linear SVMs). It is possible that thematic role information is present non-linearly within the hidden representations of LMs (see Hernandez et al., 2023; Lampinen et al., 2025). Previous research, however, has demonstrated that much conceptual information is linearly represented in hidden representations of LMs, an idea known as the “linear subspace hypothesis” (Bolukbasi et al., 2016; Vargas & Cotterell, 2020). Indeed, previous work has found that several complex concepts are present linearly in the space of hidden activations, including gender bias (Bolukbasi et al., 2016; Vargas & Cotterell, 2020), truthfulness (Li et al., 2023; Marks & Tegmark, 2023), space and time (Gurnee & Tegmark, 2023), sentiment (Tigges et al., 2023), world states (Nanda et al., 2023), and—perhaps most relevant to our study—plausibility (Kauf et al., 2023; Lee et al., 2024). Therefore, if thematic role information is available in hidden layers, it would have to differ in its representational format from these other semantic features.

A final possibility is that our study focused on the “wrong” sentence representations. We evaluated sentence representations from one token (extracting activations at [CLS] for BERT, or ‘.’ for the other LMs; but see Supplementary Materials for similar results using pooled activations across tokens in all LMs, and the representation of the verb in BERT). It is possible that thematic role information may be present in other tokens, and we leave such investigations for future work. However, we emphasize that most LMs use unidirectional attention, so the representation of any token other than the last one cannot capture the entire sentence; this leads to problems when studying structurally diverse materials such as those we used in Experiment 2, where different thematic roles can appear very early or very late in the sentence. Moreover, previous work has found that conceptual information is present in sentence representation tokens like [CLS] (e.g., Ramezani et al., 2025; Seyffarth et al., 2021).

Our study relies on probing the internal representations of LMs as a means to reveal what they know. Probing internal representations is a different approach from studying the behavior of LMs by prompting them with questions and characterizing their output. One may simply feed LMs a sentence and ask “who is the agent?”, or feed two sentences and ask whether they have matching vs. opposite thematic roles. However, such prompting makes several undesirable assumptions about LMs: that they understand the meaning of words like “agent”, that they understand how the question being asked of them relates to the sentences asked about, etc. Studying internal representations circumvents these additional “task demands” (Hu & Frank, 2024; Hu & Levy, 2023; Hu et al., 2024). In the Introduction, we further justify our use of probing instead of prompting, and we believe that our study of internal representations provides a more informative and detailed characterization of LMs than any results that would be obtained with prompting.

Unlike our characterization of LMs based on internal activity patterns, our behavioral data are based on explicit similarity ratings. Therefore, caution is warranted when descriptively comparing the two. Human data that are more easily comparable to the internal activity patterns of LMs could come from neuroimaging studies. Previous work has shown that thematic role information is indeed present in human neural representations, and meaning can be classified based on patterns of brain activity using linear classifiers similar to the ones we used here (Frankland & Greene, 2015; Wang et al., 2016).

Critically, we do not suggest that LMs are principally incapable of representing thematic roles in more human-like ways. Even though we studied LMs of varying sizes, and found that increasing model size does not appear to improve the representation of thematic roles across hidden units, it is possible that still larger sizes, or other architectures, or other training corpora, could lead models to generate such representations. In addition, models exposed to non-linguistic training, e.g., reinforcement learning from human feedback, may process thematic roles differently than those trained only on word prediction.

Nonetheless, our findings emphasize that it is vital to test the ability of LMs to process the meaning of language “per se” (in addition to their ability to exhibit non-linguistic thinking), and to do so using carefully designed and controlled materials inspired by psycholinguistics. Such studies are required for ensuring that the seemingly meaningful behavior of LMs reflects comprehension rather than non-linguistic “tricks” that have little to do with human language processing (McCoy et al., 2019). In the case of thematic roles, this approach is important because, in natural texts, these roles might be assigned based on heuristics such as plausibility (Mahowald et al., 2023). Whereas humans can use such information, in some cases they can also process thematic roles based on sentence structure alone, as suggested by our behavioral data. Our findings demonstrate that LMs can extract such information via their attention heads, but when it comes to their hidden units—commonly studied as the “ultimate” internal representation of LMs that are compared to human cognitive representations—thematic role information exerts a relatively weak influence.

## ACKNOWLEDGMENTS

We would like to thank the audiences of the 37th Annual Conference on Human Sentence Processing and the 46th Annual Meeting of the Cognitive Science Society for helpful comments on this work, as well as the anonymous reviewers for their constructive feedback. We also thank our friend, colleague, and co-author, Dr. Bryor Snefjella, who passed away in March 2023, and is dearly missed.

## AUTHOR CONTRIBUTIONS

J.M.D.: Conceptualization; Data curation; Formal analysis; Investigation; Methodology; Resources; Software; Validation; Visualization; Writing – original draft; Writing – review & editing. X.(H.)G.: Conceptualization; Investigation; Methodology; Resources; Software; Writing – review & editing. B.S.: Software. I.A.B.: Conceptualization; Methodology; Project administration; Resources; Supervision; Visualization; Writing – review & editing.

## CODE AND DATA AVAILABILITY STATEMENT

Code and data for all experiments can be found at https://osf.io/t4raz/.

## References

[bib1] Aina, L., & Linzen, T. (2021). The language model understood the prompt was ambiguous: Probing syntactic uncertainty through generation. In J. Bastings, Y. Belinkov, E. Dupoux, M. Giulianelli, D. Hupkes, Y. Pinter, & H. Sajjad (Eds.), Proceedings of the Fourth BlackboxNLP Workshop on Analyzing and Interpreting Neural Networks for NLP (pp. 42–57). Association for Computational Linguistics. 10.18653/v1/2021.blackboxnlp-1.4

[bib2] Altmann, G. T. M. (1999). Thematic role assignment in context. Journal of Memory and Language, 41, 124–145. 10.1006/jmla.1999.2640

[bib3] Ambridge, B. (2020a). Against stored abstractions: A radical exemplar model of language acquisition. First Language, 40, 509–559. 10.1177/0142723719869731

[bib4] Ambridge, B. (2020b). Abstractions made of exemplars or ‘You’re all right, and I’ve changed my mind’: Response to commentators. First Language, 40, 640–659. 10.1177/0142723720949723

[bib5] Antonello, R., Turek, J. S., Vo, V., & Huth, A. (2021). Low-dimensional structure in the space of language representations is reflected in brain responses. In M. Ranzato, A. Beygelzimer, Y. Dauphin, P. S. Liang, & J. W. Vaughan (Eds.), Proceedings of the 35th International Conference on Neural Information Processing Systems (pp. 8332–8344). Curran Associates, Inc.

[bib6] Bender, E. M., & Koller, A. (2020). Climbing towards NLU: On meaning, form, and understanding in the age of data. In D. Jurafsky, J. Chai, N. Schluter, & J. Tetreault (Eds.), Proceedings of the 58th Annual Meeting of the Association for Computational Linguistics (pp. 5185–5198). Association for Computational Linguistics. 10.18653/v1/2020.acl-main.463

[bib7] Bisk, Y., Holtzman, A., Thomason, J., Andreas, J., Bengio, Y., Chai, J., Lapata, M., Lazaridou, A., May, J., Nisnevich, A., Pinto, N., & Turian, J. (2020). Experience grounds language. arXiv. 10.48550/arXiv.2004.10151

[bib8] Blank, I. A. (2023). What are large language models supposed to model? Trends in Cognitive Sciences, 27, 987–989. 10.1016/j.tics.2023.08.006, 37659920

[bib9] Bolukbasi, T., Chang, K.-W., Zou, J. Y., Saligrama, V., & Kalai, A. T. (2016). Man is to computer programmer as woman is to homemaker? Debiasing word embeddings. In D. D. Lee, U. von Luxberg, R. Garnett, M. Sugiyama, & I. Guyon (Eds.), Proceedings of the 30th International Conference on Neural Information Processing Systems (pp. 4356–4364). Curran Associates, Inc.

[bib10] Caramazza, A., & Miceli, G. (1991). Selective impairment of thematic role assignment in sentence processing. Brain and Language, 41, 402–436. 10.1016/0093-934X(91)90164-V, 1933265

[bib11] Caramazza, A., & Zurif, E. B. (1976). Dissociation of algorithmic and heuristic processes in language comprehension: Evidence from aphasia. Brain and Language, 3, 572–582. 10.1016/0093-934X(76)90048-1, 974731

[bib12] Carreras, X., & Màrquez, L. (2005). Introduction to the CoNLL-2005 shared task: Semantic role labeling. In I. Dagan & D. Gildea (Eds.), Proceedings of the Ninth Conference on Computational Natural Language Learning (CoNLL-2005) (pp. 152–164). Association for Computational Linguistics.

[bib13] Cassani, G., Günther, F., Attanasio, G., Bianchi, F., & Marelli, M. (2023). Meaning modulations and stability in large language models: An analysis of BERT embeddings for psycholinguistic research. PsyArXiv. 10.31234/osf.io/b45ys

[bib14] Caucheteux, C., & King, J.-R. (2022). Brains and algorithms partially converge in natural language processing. Communications Biology, 5, 134. 10.1038/s42003-022-03036-1, 35173264 PMC8850612

[bib15] Chang, T. A., & Bergen, B. K. (2024). Language model behavior: A comprehensive survey. Computational Linguistics, 50, 293–350. 10.1162/coli_a_00492

[bib16] Chatterjee, A., Maher, L. M., Rothi, L. J. G., & Heilman, K. M. (1995). Asyntactic thematic role assignment: The use of a temporal-spatial strategy. Brain and Language, 49, 125–139. 10.1006/brln.1995.1024, 7648248

[bib17] Chaves, R. P., & Richter, S. N. (2021). Look at that! BERT can be easily distracted from paying attention to morphosyntax. In A. Ettinger, E. Pavlick, & B. Prickett (Eds.), Proceedings of the Society for Computation in Linguistics 2021 (pp. 28–38). Association for Computational Linguistics.

[bib18] Christiano, P. F., Leike, J., Brown, T., Martic, M., Legg, S., & Amodei, D. (2017). Deep reinforcement learning from human preferences. In U. von Luxberg, I. Guyon, S. Bengio, H. Wallach, & R. Fergus (Eds.), Proceedings of the 31st International Conference on Neural Information Processing Systems (pp. 4302–4310). Curran Associates, Inc.

[bib19] Clark, K., Khandelwal, U., Levy, O., & Manning, C. D. (2019). What does BERT look at? An analysis of BERT’s attention. arXiv. 10.48550/arXiv.1906.04341

[bib20] Cong, Y. (2024). Manner implicatures in large language models. Scientific Reports, 14, 29113. 10.1038/s41598-024-80571-3, 39582051 PMC11586417

[bib21] Contreras Kallens, P., Kristensen-McLachlan, R. D., & Christiansen, M. H. (2023). Large language models demonstrate the potential of statistical learning in language. Cognitive Science, 47, e13256. 10.1111/cogs.13256, 36840975

[bib22] Da Costa, J., & Chaves, R. (2020). Assessing the ability of transformer-based neural models to represent structurally unbounded dependencies. In A. Ettinger, G. Jarosz, & J. Pater (Eds.), Proceedings of the Society for Computation in Linguistics 2020 (pp. 12–21). Association for Computational Linguistics.

[bib23] Davies, M. (2009). The 385+ million word *Corpus of Contemporary American English* (1990–2008+): Design, architecture, and linguistic insights. International Journal of Corpus Linguistics, 14, 159–190. 10.1075/ijcl.14.2.02dav

[bib24] de Saussure, F. (2011). Course in general linguistics. Columbia University Press.

[bib25] Devlin, J., Chang, M.-W., Lee, K., & Toutanova, K. (2019). BERT: Pre-training of deep bidirectional transformers for language understanding. In J. Burstein, C. Doran, & T. Solorio (Eds.), Proceedings of the 2019 Conference of the North American Chapter of the Association for Computational Linguistics: Human Language Technologies, Volume 1 (Long and Short Papers) (pp. 4171–4186). Association for Computational Linguistics. 10.18653/v1/N19-1423

[bib26] Diego-Simón, P. J., Chemla, E., King, J.-R., & Lakretz, Y. (2025). Probing syntax in large language models: Successes and remaining challenges. arXiv. 10.48550/arXiv.2508.03211

[bib27] Diego-Simón, P. J., Orhan, P., Chemla, E., Lakretz, Y., & King, J.-R. (2026). Polar probe linearly decodes semantic structures from LLMs. arXiv. 10.48550/arXiv.2605.14125

[bib28] Dowty, D. (1991). Thematic proto-roles and argument selection. Language, 67, 547–619. 10.1353/lan.1991.0021

[bib29] Dryer, M. S. (2002). Case distinctions, rich verb agreement, and word order type (comments on Hawkins’ paper). Theoretical Linguistics, 28, 151–158. 10.1515/thli.2002.28.2.151

[bib30] Elsen, E., Odena, A., Nye, M., Taşırlar, S., Dao, T., Hawthorne, C., Moparthi, D., & Somani, A. (2023). Releasing Persimmon-8B. Adept AI. https://www.adept.ai/blog/persimmon-8b

[bib31] Ettinger, A. (2020). What BERT is not: Lessons from a new suite of psycholinguistic diagnostics for language models. Transactions of the Association for Computational Linguistics, 8, 34–48. 10.1162/tacl_a_00298

[bib32] Ettinger, A., Elgohary, A., Phillips, C., & Resnik, P. (2018). Assessing composition in sentence vector representations. In E. M. Bender, L. Derczynski, & P. Isabelle (Eds.), Proceedings of the 27th International Conference on Computational Linguistics (pp. 1790–1801). Association for Computational Linguistics.

[bib33] Ettinger, A., Elgohary, A., & Resnik, P. (2016). Probing for semantic evidence of composition by means of simple classification tasks. In Proceedings of the 1st Workshop on Evaluating Vector-Space Representations for NLP (pp. 134–139). Association for Computational Linguistics. 10.18653/v1/W16-2524

[bib34] Evanson, L., Lakretz, Y., & King, J.-R. (2023). Language acquisition: Do children and language models follow similar learning stages? arXiv. 10.48550/arXiv.2306.03586

[bib35] Fedorenko, E., Behr, M. K., & Kanwisher, N. (2011). Functional specificity for high-level linguistic processing in the human brain. Proceedings of the National Academy of Sciences of the United States of America, 108, 16428–16433. 10.1073/pnas.1112937108, 21885736 PMC3182706

[bib36] Fedorenko, E., Blank, I. A., Siegelman, M., & Mineroff, Z. (2020). Lack of selectivity for syntax relative to word meanings throughout the language network. Cognition, 203, 104348. 10.1016/j.cognition.2020.104348, 32569894 PMC7483589

[bib37] Fedorenko, E., Ivanova, A. A., & Regev, T. I. (2024). The language network as a natural kind within the broader landscape of the human brain. Nature Reviews Neuroscience, 25, 289–312. 10.1038/s41583-024-00802-4, 38609551 PMC13222024

[bib38] Fedorenko, E., Scott, T. L., Brunner, P., Coon, W. G., Pritchett, B., Schalk, G., & Kanwisher, N. (2016). Neural correlate of the construction of sentence meaning. Proceedings of the National Academy of Sciences of the United States of America, 113, E6256–E6262. 10.1073/pnas.1612132113, 27671642 PMC5068329

[bib39] Fedorenko, E., & Varley, R. (2016). Language and thought are not the same thing: Evidence from neuroimaging and neurological patients. Annals of the New York Academy of Sciences, 1369, 132–153. 10.1111/nyas.13046, 27096882 PMC4874898

[bib40] Fenk-Oczlon, G., & Fenk, A. (2008). Complexity trade-offs between the subsystems of language. In M. Miestamo, K. Sinnemäki, & F. Karlsson (Eds.), Language complexity: Typology, contact, change (pp. 43–65). John Benjamins Publishing Company. 10.1075/slcs.94.05fen

[bib65] Ferreira, F., & Lowder, M. W. (2016). Prediction, information structure, and good-enough language processing. In B. H. Ross (Ed.), Psychology of learning and motivation (Vol. 65, pp. 217–247). Elsevier. 10.1016/bs.plm.2016.04.002

[bib41] Fillmore, C. J. (1968). The case for case. In E. Bach & R. T. Harms (Eds.), Universals in linguistic theory (pp. 1–88). Holt, Rinehart, and Winston.

[bib42] Firth, J. R. (1962). Studies in linguistic analysis: Special volume of the Philological Society. Blackwell.

[bib44] Frankland, S. M., & Greene, J. D. (2015). An architecture for encoding sentence meaning in left mid-superior temporal cortex. Proceedings of the National Academy of Sciences of the United States of America, 112, 11732–11737. 10.1073/pnas.1421236112, 26305927 PMC4577152

[bib43] Frankland, S. M., & Greene, J. D. (2020). Two ways to build a thought: Distinct forms of compositional semantic representation across brain regions. Cerebral Cortex, 30, 3838–3855. 10.1093/cercor/bhaa001, 32279078

[bib45] Futrell, R., & Mahowald, K. (2026). How linguistics learned to stop worrying and love the language models. Behavioral and Brain Sciences, 49, e198. 10.1017/S0140525X2510112X, 40702714

[bib46] Gennari, S. P., & MacDonald, M. C. (2008). Semantic indeterminacy in object relative clauses. Journal of Memory and Language, 58, 161–187. 10.1016/j.jml.2007.07.004, 19724662 PMC2735264

[bib47] Glockner, M., Shwartz, V., & Goldberg, Y. (2018). Breaking NLI systems with sentences that require simple lexical inferences. arXiv. 10.48550/arXiv.1805.02266

[bib49] Goldberg, A. E. (1995). Constructions: A construction grammar approach to argument structure. University of Chicago Press.

[bib48] Goldberg, A. E. (2019). Explain me this: Creativity, competition, and the partial productivity of constructions. Princeton University Press. 10.1515/9780691183954

[bib50] Grand, G., Blank, I. A., Pereira, F., & Fedorenko, E. (2022). Semantic projection recovers rich human knowledge of multiple object features from word embeddings. Nature Human Behaviour, 6, 975–987. 10.1038/s41562-022-01316-8, 35422527 PMC10349641

[bib51] Greenberg, J. H. (1963). Some universals of grammar with particular reference to the order of meaningful elements. In J. H. Greenberg (Ed.), Universals of language (2nd ed., pp. 73–113). MIT Press.

[bib52] Gurnee, W., & Tegmark, M. (2023). Language models represent space and time. arXiv. 10.48550/arXiv.2310.02207

[bib53] Harris, Z. S. (1954). Distributional structure. WORD, 10, 146–162. 10.1080/00437956.1954.11659520

[bib54] Hartshorne, J., & Snedeker, J. (2026). Conceptual nativism: The origins of language are in the structure of thought. PsyArXiv. 10.31234/osf.io/ur3gj_v1

[bib55] Hebart, M. N., Zheng, C. Y., Pereira, F., & Baker, C. I. (2020). Revealing the multidimensional mental representations of natural objects underlying human similarity judgements. Nature Human Behaviour, 4, 1173–1185. 10.1038/s41562-020-00951-3, 33046861 PMC7666026

[bib56] Hernandez, E., Sharma, A. S., Haklay, T., Meng, K., Wattenberg, M., Andreas, J., Belinkov, Y., & Bau, D. (2023). Linearity of relation decoding in transformer language models. arXiv. 10.48550/arXiv.2308.09124

[bib57] Hosseini, E. A., Schrimpf, M., Zhang, Y., Bowman, S., Zaslavsky, N., & Fedorenko, E. (2022). Artificial neural network language models align neurally and behaviorally with humans even after a developmentally realistic amount of training. bioRxiv. 10.1101/2022.10.04.510681 PMC11025646 38645622

[bib58] Hu, J., & Frank, M. C. (2024). Auxiliary task demands mask the capabilities of smaller language models. arXiv. 10.48550/arXiv.2404.02418

[bib59] Hu, J., Gauthier, J., Qian, P., Wilcox, E., & Levy, R. P. (2020). A systematic assessment of syntactic generalization in neural language models. arXiv. 10.48550/arXiv.2005.03692

[bib60] Hu, J., & Levy, R. (2023). Prompting is not a substitute for probability measurements in large language models. In H. Bouamor, J. Pino, & K. Bali (Eds.), Proceedings of the 2023 Conference on Empirical Methods in Natural Language Processing (pp. 5040–5060). Association for Computational Linguistics. 10.18653/v1/2023.emnlp-main.306

[bib61] Hu, J., Mahowald, K., Lupyan, G., Ivanova, A., & Levy, R. (2024). Language models align with human judgments on key grammatical constructions. Proceedings of the National Academy of Sciences of the United States of America, 121, e2400917121. 10.1073/pnas.2400917121, 39186652 PMC11388428

[bib62] Hu, J., Wilcox, E. G., Song, S., Mahowald, K., & Levy, R. P. (2025). What can string probability tell us about grammaticality? arXiv. 10.48550/arXiv.2510.16227

[bib63] Huang, K.-J., Arehalli, S., Kugemoto, M., Muxica, C., Prasad, G., Dillon, B., & Linzen, T. (2024). Large-scale benchmark yields no evidence that language model surprisal explains syntactic disambiguation difficulty. Journal of Memory and Language, 137, 104510. 10.1016/j.jml.2024.104510

[bib64] Iaia, C., Choksi, B., Wiebers, E., Roig, G., & Fiebach, C. J. (2025). The representational alignment between humans and language models is implicitly driven by a concreteness effect. arXiv. 10.48550/arXiv.2505.15682

[bib66] Ivanova, A. A., Kauf, C., Gao, R., She, J. S., Kean, H. H., Goldhaber, T., Nieto-Castañón, A., Varley, R., Kanwisher, N., & Fedorenko, E. (2025). Semantic reasoning takes place largely outside the language network. bioRxiv. 10.64898/2025.12.07.692873

[bib156] Ivanova, A. A., Mineroff, Z., Zimmerer, V., Kanwisher, N., Varley, R., & Fedorenko, E. (2021). The language network is recruited but not required for nonverbal event semantics. Neurobiology of Language, 2(2), 176–201. 10.1162/nol_a_00030, 37216147 PMC10158592

[bib67] Jackendoff, R. (2012). A user’s guide to thought and meaning. Oxford University Press.

[bib68] Jackendoff, R., & Wittenberg, E. (2017). Linear grammar as a possible stepping-stone in the evolution of language. Psychonomic Bulletin & Review, 24, 219–224. 10.3758/s13423-016-1073-y, 27368633

[bib69] Jackendoff, R. S. (1972). Semantic interpretation in generative grammar. MIT Press.

[bib70] Jozwik, K. M., Kriegeskorte, N., Storrs, K. R., & Mur, M. (2017). Deep convolutional neural networks outperform feature-based but not categorical models in explaining object similarity judgments. Frontiers in Psychology, 8, 1726. 10.3389/fpsyg.2017.01726, 29062291 PMC5640771

[bib71] Kauf, C., Ivanova, A. A., Rambelli, G., Chersoni, E., She, J. S., Chowdhury, Z., Fedorenko, E., & Lenci, A. (2023). Event knowledge in large language models: The gap between the impossible and the unlikely. Cognitive Science, 47(11), e13386. 10.1111/cogs.13386, 38009752

[bib72] Kim, N., & Schuster, S. (2023). Entity tracking in language models. arXiv. 10.48550/arXiv.2305.02363

[bib73] Kiparsky, P. (1997). The rise of positional licensing. In A. van Kemenade & N. Vincent (Eds.), Parameters of morphosyntactic change (pp. 460–494). Cambridge University Press.

[bib74] Kodali, P., Goel, A., Asapu, L., Bonagiri, V. K., Govil, A., Choudhury, M., Kumaraguru, P., & Shrivastava, M. (2025). From human judgements to predictive models: Unravelling acceptability in code-mixed sentences. ACM Transactions on Asian and Low-Resource Language Information Processing, 24, 1–31. 10.1145/3748312

[bib75] Koplenig, A., Meyer, P., Wolfer, S., & Müller-Spitzer, C. (2017). The statistical trade-off between word order and word structure – Large-scale evidence for the principle of least effort. PLoS One, 12(3), e0173614. 10.1371/journal.pone.0173614, 28282435 PMC5345836

[bib76] Kovaleva, O., Romanov, A., Rogers, A., & Rumshisky, A. (2019). Revealing the dark secrets of BERT. arXiv. 10.48550/arXiv.1908.08593

[bib77] Kriegeskorte, N., Mur, M., & Bandettini, P. (2008). Representational similarity analysis—Connecting the branches of systems neuroscience. Frontiers in Systems Neuroscience, 2, 4. 10.3389/neuro.06.004.2008, 19104670 PMC2605405

[bib78] Lampinen, A. K., Chan, S. C. Y., Li, Y., & Hermann, K. (2025). Representation biases: Will we achieve complete understanding by analyzing representations? arXiv. 10.48550/arXiv.2507.22216

[bib79] Lau, J. H., Armendariz, C., Lappin, S., Purver, M., & Shu, C. (2020). How furiously can colorless green ideas sleep? Sentence acceptability in context. Transactions of the Association for Computational Linguistics, 8, 296–310. 10.1162/tacl_a_00315

[bib80] Lee, E.-K. R., Nair, S., & Feldman, N. (2024). A psycholinguistic evaluation of language models’ sensitivity to argument roles. arXiv. 10.48550/arXiv.2410.16139

[bib81] Lee, E.-K. R., & Phillips, C. (2025). Argument role sensitivity in real-time sentence processing: Evidence from a hybrid comprehension and production task. Cognition, 264, 106255. 10.1016/j.cognition.2025.106255, 40675055

[bib82] Levin, B., & Hovav, M. R. (2005). Argument realization. Cambridge University Press. 10.1017/CBO9780511610479

[bib84] Levshina, N. (2020). Efficient trade-offs as explanations in functional linguistics: Some problems and an alternative proposal. Revista da Abralin, 19, 50–78. 10.25189/rabralin.v19i3.1728

[bib83] Levshina, N. (2021). Cross-linguistic trade-offs and causal relationships between cues to grammatical subject and object, and the problem of efficiency-related explanations. Frontiers in Psychology, 12, 648200. 10.3389/fpsyg.2021.648200, 34322056 PMC8311235

[bib85] Li, K., Patel, O., Viégas, F., Pfister, H., & Wattenberg, M. (2023). Inference-time intervention: Eliciting truthful answers from a language model. In A. Oh, T. Naumann, A. Globerson, K. Saenko, M. Hardt, & S. Levine (Eds.), Proceedings of the 37th International Conference on Neural Information Processing Systems (pp. 41451–41530). Curran Associates, Inc.

[bib86] Linzen, T., & Baroni, M. (2021). Syntactic structure from deep learning. Annual Review of Linguistics, 7, 195–212. 10.1146/annurev-linguistics-032020-051035

[bib87] Liu, Q., van Paridon, J., & Lupyan, G. (2025). Learning about color from language. Communications Psychology, 3(1), 60. 10.1038/s44271-025-00230-9, 40229351 PMC11997174

[bib88] Lu, J., Merchan, J., Wang, L., & Degen, J. (2024). Can syntactic log-odds ratio predict acceptability and satiation? In R. Futrell, C. Mayer, & N. Zaslavsky (Eds.), Proceedings of the Society for Computation in Linguistics 2024 (pp. 10–19). Association for Computational Linguistics.

[bib89] Mahner, F. P., Muttenthaler, L., Güçlü, U., & Hebart, M. N. (2025). Dimensions underlying the representational alignment of deep neural networks with humans. Nature Machine Intelligence, 7, 848–859. 10.1038/s42256-025-01041-7, 40567352 PMC12185338

[bib90] Mahowald, K., Diachek, E., Gibson, E., Fedorenko, E., & Futrell, R. (2023). Grammatical cues to subjecthood are redundant in a majority of simple clauses across languages. Cognition, 241, 105543. 10.1016/j.cognition.2023.105543, 37713956

[bib91] Mahowald, K., Ivanova, A. A., Blank, I. A., Kanwisher, N., Tenenbaum, J. B., & Fedorenko, E. (2024). Dissociating language and thought in large language models. Trends in Cognitive Sciences, 28, 517–540. 10.1016/j.tics.2024.01.011, 38508911 PMC11416727

[bib92] Manning, C. D., Clark, K., Hewitt, J., Khandelwal, U., & Levy, O. (2020). Emergent linguistic structure in artificial neural networks trained by self-supervision. Proceedings of the National Academy of Sciences of the United States of America, 117, 30046–30054. 10.1073/pnas.1907367117, 32493748 PMC7720155

[bib93] Marks, S., & Tegmark, M. (2023). The geometry of truth: Emergent linear structure in large language model representations of true/false datasets. arXiv. 10.48550/arXiv.2310.06824

[bib94] Marvin, R., & Linzen, T. (2018). Targeted syntactic evaluation of language models. arXiv. 10.48550/arXiv.1808.09031

[bib95] May, C., Wang, A., Bordia, S., Bowman, S. R., & Rudinger, R. (2019). On measuring social biases in sentence encoders. arXiv. 10.48550/arXiv.1903.10561

[bib96] McCoy, R. T., Pavlick, E., & Linzen, T. (2019). Right for the wrong reasons: Diagnosing syntactic heuristics in natural language inference. arXiv. 10.48550/arXiv.1902.01007

[bib97] McCoy, R. T., Smolensky, P., Linzen, T., Gao, J., & Celikyilmaz, A. (2023). How much do language models copy from their training data? Evaluating linguistic novelty in text generation using RAVEN. Transactions of the Association for Computational Linguistics, 11, 652–670. 10.1162/tacl_a_00567

[bib98] McGee, T. A., Zhang, Y., & Blank, I. A. (2026). Evidence against syntactic encapsulation in large language models. Cognitive Science, 50, e70187. 10.1111/cogs.70187, 41805029 PMC12973484

[bib99] Merkx, D., & Frank, S. L. (2020). Human sentence processing: Recurrence or attention? arXiv. 10.48550/arXiv.2005.09471

[bib100] Merrill, W., Goldberg, Y., Schwartz, R., & Smith, N. A. (2021). Provable limitations of acquiring meaning from ungrounded form: What will future language models understand? Transactions of the Association for Computational Linguistics, 9, 1047–1060. 10.1162/tacl_a_00412

[bib101] Muttenthaler, L., Linhardt, L., Dippel, J., Vandermeulen, R. A., Hermann, K., Lampinen, A., & Kornblith, S. (2023). Improving neural network representations using human similarity judgments. In A. Oh, T. Naumann, A. Globerson, K. Saenko, M. Hardt, & S. Levine (Eds.), Proceedings of the 37th International Conference on Neural Information Processing Systems (pp. 50978–51007). Curran Associates, Inc.

[bib102] Nakamura, M., Momma, S., Sakai, H., & Phillips, C. (2024). Task and timing effects in argument role sensitivity: Evidence from production, EEG, and computational modeling. Cognitive Science, 48(12), e70023. 10.1111/cogs.70023, 39625951 PMC11614320

[bib103] Nanda, N., Lee, A., & Wattenberg, M. (2023). Emergent linear representations in world models of self-supervised sequence models. arXiv. 10.48550/arXiv.2309.00941

[bib104] Nicolas, G., & Caliskan, A. (2024). Directionality and representativeness are differentiable components of stereotypes in large language models. PNAS Nexus, 3, pgae493. 10.1093/pnasnexus/pgae493, 39588319 PMC11586767

[bib105] Norman, K. A., Polyn, S. M., Detre, G. J., & Haxby, J. V. (2006). Beyond mind-reading: Multi-voxel pattern analysis of fMRI data. Trends in Cognitive Sciences, 10(9), 424–430. 10.1016/j.tics.2006.07.005, 16899397

[bib106] Oh, B.-D., & Schuler, W. (2022). Entropy- and distance-based predictors from GPT-2 attention patterns predict reading times over and above GPT-2 surprisal. arXiv. 10.48550/arXiv.2212.11185

[bib107] OpenAI. (2022). ChatGPT: Optimizing language models for dialogue. OpenAI. https://openai.com/index/chatgpt/

[bib108] Ouyang, L., Wu, J., Jiang, X., Almeida, D., Wainwright, C., Mishkin, P., Zhang, C., Agarwal, S., Slama, K., Ray, A., Schulman, J., Hilton, J., Kelton, F., Miller, L., Simens, M., Askell, A., Welinder, P., Christiano, P. F., Leike, J., & Lowe, R. (2022). Training language models to follow instructions with human feedback. In S. Koyejo, S. Mohamed, A. Agarwal, D. Belgrave, K. Cho, & A. Oh (Eds.), Proceedings of the 36th International Conference on Neural Information Processing Systems (pp. 27730–27744). Curran Associates, Inc.

[bib109] Papadimitriou, I., Futrell, R., & Mahowald, K. (2022). When classifying grammatical role, BERT doesn’t care about word order … except when it matters. arXiv. 10.48550/arXiv.2203.06204

[bib110] Pavlick, E. (2022). Semantic structure in deep learning. Annual Review of Linguistics, 8, 447–471. 10.1146/annurev-linguistics-031120-122924

[bib111] Pennington, J., Socher, R., & Manning, C. D. (2014). GloVe: Global vectors for word representation. In A. Moschitti, B. Pang, & W. Daelemans (Eds.), Proceedings of the 2014 Conference on Empirical Methods in Natural Language Processing (EMNLP) (pp. 1532–1543). Association for Computational Linguistics. 10.3115/v1/D14-1162

[bib112] Piantadosi, S. (2023). Modern language models refute Chomsky’s approach to language. Lingbuzz. https://lingbuzz.net/lingbuzz/007180

[bib113] Piantadosi, S. T., & Hill, F. (2022). Meaning without reference in large language models. arXiv. 10.48550/arXiv.2208.02957

[bib114] Pinker, S. (2009). Language learnability and language development: With new commentary by the author. Harvard University Press. 10.4159/9780674042179

[bib115] Pradhan, S., Moschitti, A., Xue, N., Ng, H. T., Björkelund, A., Uryupina, O., Zhang, Y., & Zhong, Z. (2013). Towards robust linguistic analysis using OntoNotes. In J. Hockenmaier & S. Riedel (Eds.), Proceedings of the Seventeenth Conference on Computational Natural Language Learning (pp. 143–152). Association for Computational Linguistics.

[bib116] Proietti, M., Lebani, G. E., & Lenci, A. (2024). On the proto-role properties inferred by transformer language models. Lingue e Linguaggio, 23, 111–140. 10.1418/113930

[bib117] Proietti, M., Lebani, G. E., & Lenci, A. (2022). Does BERT recognize an agent? Modeling Dowty’s proto-roles with contextual embeddings. In N. Calzolari, C.-R. Huang, H. Kim, J. Pustejovsky, L. Wanner, K.-S. Choi, P.-M. Ryu, H.-H. Chen, L. Donatelli, H. Ji, S. Kurohashi, P. Paggio, N. Xue, S. Kim, Y. Hahm, Z. He, T. K. Lee, E. Santus, F. Bond, & S.-H. Na (Eds.), Proceedings of the 29th International Conference on Computational Linguistics (pp. 4101–4112). International Committee on Computational Linguistics.

[bib118] Radford, A., Wu, J., Child, R., Luan, D., Amodei, D., & Sutskever, I. (2019). Language models are unsupervised multitask learners. OpenAI Blog. https://cdn.openai.com/better-language-models/language_models_are_unsupervised_multitask_learners.pdf

[bib119] Ramezani, P., Schilling, A., & Krauss, P. (2025). Analysis of argument structure constructions in the large language model BERT. Frontiers in Artificial Intelligence, 8, 1477246. 10.3389/frai.2025.1477246, 39959915 PMC11825518

[bib120] Regev, T. I., Casto, C., Hosseini, E. A., Adamek, M., Ritaccio, A. L., Willie, J. T., Brunner, P., & Fedorenko, E. (2024). Neural populations in the language network differ in the size of their temporal receptive windows. Nature Human Behaviour, 8(10), 1924–1942. 10.1038/s41562-024-01944-2, 39187713

[bib121] Rissman, L., & Majid, A. (2019). Thematic roles: Core knowledge or linguistic construct? Psychonomic Bulletin & Review, 26, 1850–1869. 10.3758/s13423-019-01634-5, 31290008 PMC6863944

[bib122] Rosenman, S., Jacovi, A., & Goldberg, Y. (2020). Exposing shallow heuristics of relation extraction models with challenge data. arXiv. 10.48550/arXiv.2010.03656

[bib123] Ryskin, R., & Nieuwland, M. S. (2023). Prediction during language comprehension: What is next? Trends in Cognitive Sciences, 27, 1032–1052. 10.1016/j.tics.2023.08.003, 37704456 PMC11614350

[bib124] Schrimpf, M., Blank, I. A., Tuckute, G., Kauf, C., Hosseini, E. A., Kanwisher, N., Tenenbaum, J. B., & Fedorenko, E. (2021). The neural architecture of language: Integrative modeling converges on predictive processing. Proceedings of the National Academy of Sciences of the United States of America, 118(45), e2105646118. 10.1073/pnas.2105646118, 34737231 PMC8694052

[bib125] Schuster, S., & Linzen, T. (2022). When a sentence does not introduce a discourse entity, transformer-based models still sometimes refer to it. arXiv. 10.48550/arXiv.2205.03472

[bib126] Seyffarth, E., Samih, Y., Kallmeyer, L., & Sajjad, H. (2021). Implicit representations of event properties within contextual language models: Searching for “causativity neurons.” In S. Zarrieß, J. Bos, R. van Noord, & L. Abzianidze (Eds.), Proceedings of the 14th International Conference on Computational Semantics (IWCS) (pp. 110–120). Association for Computational Linguistics.

[bib157] Silver, N. C., & Dunlap, W. P. (1987). Averaging correlation coefficients: Should Fisher’s *z* transformation be used? Journal of Applied Psychology, 72(1), 146–148. 10.1037/0021-9010.72.1.146

[bib158] Singh, A., Fry, A., Perelman, A., Tart, A., Ganesh, A., El-Kishky, A., McLaughlin, A., Low, A., Ostrow, A. J., Ananthram, A., Nathan, A., Luo, A., Helyar, A., Madry, A., Efremov, A., Spyra, A., Baker-Whitcomb, A., Beutel, A., Karpenko, A., … Wang, Z. (2026). OpenAI GPT-5 System Card. arXiv. 10.48550/arXiv.2601.03267

[bib127] Sinha, K., Jia, R., Hupkes, D., Pineau, J., Williams, A., & Kiela, D. (2021). Masked language modeling and the distributional hypothesis: Order word matters pre-training for little. arXiv. 10.48550/arXiv.2104.06644

[bib128] Sinha, K., Sodhani, S., Dong, J., Pineau, J., & Hamilton, W. L. (2019). CLUTRR: A diagnostic benchmark for inductive reasoning from text. arXiv. 10.48550/arXiv.1908.06177

[bib129] Sinnemäki, K. (2008). Complexity trade-offs in core argument marking. In M. Miestamo, K. Sinnemäki, & F. Karlsson (Eds.), Language complexity: Typology, contact, change (pp. 67–88). John Benjamins Publishing Company. 10.1075/slcs.94.06sin

[bib130] Srivastava, A., Rastogi, A., Rao, A., Shoeb, A. A. M., Abid, A., Fisch, A., Brown, A. R., Santoro, A., Gupta, A., Garriga-Alonso, A., Kluska, A., Lewkowycz, A., Agarwal, A., Power, A., Ray, A., Warstadt, A., Kocurek, A. W., Safaya, A., Tazarv, A., … Wu, Z. (2022). Beyond the Imitation Game: Quantifying and extrapolating the capabilities of language models. arXiv. 10.48550/arXiv.2206.04615

[bib131] Takahashi, S., Sasaki, M., Takeda, K., & Oizumi, M. (2025). Investigating fine- and coarse-grained structural correspondences between deep neural networks and human object image similarity judgments using unsupervised alignment. arXiv. 10.48550/arXiv.2505.16419 41151525

[bib132] Tang, J., Du, M., Vo, V. A., Lal, V., & Huth, A. G. (2023). Brain encoding models based on multimodal transformers can transfer across language and vision. In A. Oh, T. Naumann, A. Globerson, K. Saenko, M. Hardt, & S. Levine (Eds.), Proceedings of the 37th International Conference on Neural Information Processing Systems (pp. 29654–29666). Curran Associates, Inc. PMC11250991 39015152

[bib133] Tarigopula, P., Fairhall, S. L., Bavaresco, A., Truong, N., & Hasson, U. (2023). Improved prediction of behavioral and neural similarity spaces using pruned DNNs. Neural Networks, 168, 89–104. 10.1016/j.neunet.2023.08.049, 37748394

[bib134] Tenney, I., Das, D., & Pavlick, E. (2019). BERT rediscovers the classical NLP pipeline. arXiv. 10.48550/arXiv.1905.05950

[bib135] Tenney, I., Xia, P., Chen, B., Wang, A., Poliak, A., McCoy, R. T., Kim, N., Van Durme, B., Bowman, S. R., Das, D., & Pavlick, E. (2019). What do you learn from context? Probing for sentence structure in contextualized word representations. arXiv. 10.48550/arXiv.1905.06316

[bib136] Tigges, C., Hollinsworth, O. J., Geiger, A., & Nanda, N. (2023). Linear representations of sentiment in large language models. arXiv. 10.48550/arXiv.2310.15154

[bib137] Timkey, W., & van Schijndel, M. (2021). All bark and no bite: Rogue dimensions in transformer language models obscure representational quality. arXiv. 10.48550/arXiv.2109.04404

[bib139] Tomasello, M. (1992). First verbs: A case study of early grammatical development. Cambridge University Press. 10.1017/CBO9780511527678

[bib138] Tomasello, M. (2003). Constructing a language: A usage-based theory of language acquisition. Harvard University Press. 10.2307/j.ctv26070v8

[bib140] Touvron, H., Martin, L., Stone, K., Albert, P., Almahairi, A., Babaei, Y., Bashlykov, N., Batra, S., Bhargava, P., Bhosale, S., Bikel, D., Blecher, L., Ferrer, C. C., Chen, M., Cucurull, G., Esiobu, D., Fernandes, J., Fu, J., Fu, W., … Scialom, T. (2023). Llama 2: Open foundation and fine-tuned chat models. arXiv. 10.48550/arXiv.2307.09288

[bib141] Tuckute, G., Sathe, A., Srikant, S., Taliaferro, M., Wang, M., Schrimpf, M., Kay, K., & Fedorenko, E. (2024). Driving and suppressing the human language network using large language models. Nature Human Behaviour, 8(3), 544–561. 10.1038/s41562-023-01783-7, 38172630

[bib142] Ullman, T. (2023). Large language models fail on trivial alterations to theory-of-mind tasks. arXiv. 10.48550/arXiv.2302.08399

[bib143] Utsumi, A. (2020). Exploring what is encoded in distributional word vectors: A neurobiologically motivated analysis. Cognitive Science, 44(6), e12844. 10.1111/cogs.12844, 32458523

[bib144] van Schijndel, M., & Linzen, T. (2021). Single-stage prediction models do not explain the magnitude of syntactic disambiguation difficulty. Cognitive Science, 45(6), e12988. 10.1111/cogs.12988, 34170031

[bib145] Vargas, F., & Cotterell, R. (2020). Exploring the linear subspace hypothesis in gender bias mitigation. arXiv. 10.48550/arXiv.2009.09435

[bib146] Wang, A., Singh, A., Michael, J., Hill, F., Levy, O., & Bowman, S. R. (2018). GLUE: A multi-task benchmark and analysis platform for natural language understanding. arXiv. 10.48550/arXiv.1804.07461

[bib147] Wang, J., Cherkassky, V. L., Yang, Y., Chang, K.-M. K., Vargas, R., Diana, N., & Just, M. A. (2016). Identifying thematic roles from neural representations measured by functional magnetic resonance imaging. Cognitive Neuropsychology, 33(3–4), 257–264. 10.1080/02643294.2016.1182480, 27314175

[bib148] Wang, K., Variengien, A., Conmy, A., Shlegeris, B., & Steinhardt, J. (2022). Interpretability in the wild: A circuit for indirect object identification in GPT-2 small. arXiv. 10.48550/arXiv.2211.00593

[bib149] Warstadt, A., & Bowman, S. R. (2020). Linguistic analysis of pretrained sentence encoders with acceptability judgments. arXiv. 10.48550/arXiv.1901.03438

[bib150] Warstadt, A., Parrish, A., Liu, H., Mohananey, A., Peng, W., Wang, S.-F., & Bowman, S. R. (2020). BLiMP: The Benchmark of Linguistic Minimal Pairs for English. Transactions of the Association for Computational Linguistics, 8, 377–392. 10.1162/tacl_a_00321

[bib151] Weissweiler, L., Hofmann, V., Köksal, A., & Schütze, H. (2022). The better your syntax, the better your semantics? Probing pretrained language models for the English comparative correlative. arXiv. 10.48550/arXiv.2210.13181

[bib152] Wilcox, E. G., Gauthier, J., Hu, J., Qian, P., & Levy, R. (2020). On the predictive power of neural language models for human real-time comprehension behavior. arXiv. 10.48550/arXiv.2006.01912

[bib153] Wilcox, E. G., Vani, P., & Levy, R. P. (2021). A targeted assessment of incremental processing in neural language models and humans. In C. Zong, F. Xia, W. Li, & R. Navigli (Eds.), Proceedings of the 59th Annual Meeting of the Association for Computational Linguistics and the 11th International Joint Conference on Natural Language Processing (Volume 1: Long Papers) (pp. 939–952). Association for Computational Linguistics. 10.18653/v1/2021.acl-long.76

[bib154] Wu, Z., Qiu, L., Ross, A., Akyürek, E., Chen, B., Wang, B., Kim, N., Andreas, J., & Kim, Y. (2023). Reasoning or reciting? Exploring the capabilities and limitations of language models through counterfactual tasks. arXiv. 10.48550/arXiv.2307.02477

[bib155] Ziegler, J., & Snedeker, J. (2018). How broad are thematic roles? Evidence from structural priming. Cognition, 179, 221–240. 10.1016/j.cognition.2018.06.019, 30064653

